# Framework for evaluating explainable AI in antimicrobial drug discovery

**DOI:** 10.1186/s13321-026-01200-x

**Published:** 2026-05-04

**Authors:** Abdulmujeeb T. Onawole, Mark A. T. Blaskovich, Johannes Zuegg

**Affiliations:** 1 Centre for Superbug Solutions, Institute for Molecular Bioscience, The University of Queensland, Brisbane, QLD Australia; 2 ARC Centre for Agricultural and Environmental Solutions to Antimicrobial Resistance, Institute for Molecular Bioscience, The University of Queensland, Brisbane, QLD Australia

**Keywords:** Machine learning, Deep Neural Networks, Explainable AI, Antibacterial prediction, Drug development, Structure–activity relationship, Chemoinformatic, Molecular decomposition

## Abstract

**Abstract:**

Explainable artificial intelligence (XAI) methods for molecular property prediction lack standardized evaluation criteria, preventing widespread deployment in drug development and hit optimisation, where proper understanding of structure–activity relationship is essential. We developed an evaluation framework for XAI using fragment-based explainability tests to compare XAI with different molecular representation and challenge the different XAI approaches for proper explanation of activity cliffs. The evaluation methods include essential scaffold recognition, scaffold sensitivity and substructure specificity for explaining activity cliff, and technical evaluation on model robustness and consistency. Using a curated dataset of antibiotic molecules we established three XAI models with fundamentally different molecular representation: Random Forest on chemical features using SHAP, CNN on sequence-based SMILES using token occlusion, and RGCN on molecular graphs with substructure masking. Together with detailed case study, we evaluated the explainability behaviours and quality of the different XAI approaches and highlighted their limitations. While all XAI approaches displayed good predictive and scaffold recognition capabilities, and comparable robustness and consistency, they displayed quite different explainability behaviour for activity cliffs, revealing their different utility for medicinal chemistry.

**Scientific contribution:**

A.T.O. performed the study, A.T.O and J.Z. contributed to the concept of the study and wrote the original manuscript. All authors reviewed the manuscript.

**Supplementary Information:**

The online version contains supplementary material available at 10.1186/s13321-026-01200-x.

## Introduction

Antimicrobial resistance (AMR) constitutes a significant global health crisis, threatening healthcare advancements by limiting effective therapeutic options against multidrug-resistant pathogens [1]. The rapid integration of artificial intelligence (AI) into drug discovery promises accelerated identification and optimization of novel antimicrobial agents [2, 3]. While AI models have demonstrated remarkable success in predicting biological activities and identifying potential drug candidates from vast chemical libraries, their "black box" nature prevents them in providing practical guidance about chemical reasons for their predictions. For medicinal chemists working to optimize drug candidates, understanding which molecular features drive activity is essential for rational drug design. Consequently, the adoption of AI in medicinal chemistry workflows has been slower than anticipated, emphasizing the need for more explainable and transparent AI methods.

Explainable AI (XAI) techniques have emerged to address this transparency challenge by providing chemically meaningful rationales for AI-driven predictions [4]. These methods aim to bridge the gap between computational predictions and actionable chemical insights that medicinal chemists can use to guide molecular optimization. While predictive AI models are able to use a range of well-established performance criteria to evaluate the accuracy and limitations of the prediction, evaluating the accuracy of explainable AI models is less defined. This opacity becomes particularly problematic in drug development, where explanations that appear plausible but are actually unfaithful, incoherent, or unstable can lead medicinal chemists to make incorrect optimization decisions, potentially resulting in failed drug candidates and wasted resources [5–7].

Current XAI approaches vary widely in complexity and methodology, from popular methods like SHAP [8] and LIME [9] to perturbation-based techniques [10] and substructure masking approaches [11]. While recent reviews have highlighted the potential of XAI methods in drug discovery [12, 13], empirical comparisons of these approaches remain limited, particularly in evaluating whether their explanations align with established medicinal chemistry principles.

A fundamental challenge in the application of XAI in the prediction of molecular properties is the lack of evaluation frameworks for assessing the accuracy of molecular explanations, between different model approaches or even between different datasets. Without systematic evaluation frameworks, medicinal chemists cannot determine which XAI models provide trustworthy insights for rational drug design decisions, perpetuating the black box problem that limits AI adoption in pharmaceutical research.

Recent work has demonstrated meaningful progress toward systematic XAI evaluation in chemistry, particularly through benchmarks that assess whether models can correctly identify known structural motifs or atom–bond patterns linked to molecular properties. For example, quantitative GNN–XAI benchmarks have been developed to measure how effectively attribution methods recover known toxicophores, mutagenicity alerts, and other curated substructures [14], and the B-XAIC dataset provides atom- and edge-level labels for identifying chemical motifs such as halogens, indoles, PAINS alerts, and complex ring patterns across a large molecular corpus [15]. These advances offer foundations for evaluating molecular explanations, though they mainly focus on motif-recovery accuracy, rather than medicinal-chemistry reasoning needed to assess the scaffold contributions, context-dependent structure–activity effects, or attribution stability across training conditions.

To address these concerns about explanation reliability and prevent the deployment of potentially misleading XAI systems in the drug development process, our evaluation methodology focuses on four essential capabilities that any XAI method should demonstrate for reliable deployment in drug discovery. We developed a four-tier evaluation framework assessing scaffold recognition, context sensitivity, internal consistency, and model independence across well-characterized classes of antibiotic compounds. This validation approach uses established medicinal chemistry benchmarks where structure–activity relationships (SAR) have been extensively characterized. Three major antibiotic classes were selected as validation benchmarks: β-lactams – inhibitors of penicillin-binding proteins (PBPs), transpeptidases essential for peptidoglycan cross-linking during cell wall synthesis [16], fluoroquinolones—inhibitors of DNA gyrase (GyrA/GyrB) and topoisomerase IV (ParC/ParE), blocking DNA replication and transcription [17], and oxazolidinones – binding to the 50S ribosomal subunit at the peptidyl transferase centre, inhibiting formation of the 70S initiation complex and blocking bacterial protein synthesis [18]. These classes represent different mode of actions, with well-defined pharmacophores and SAR, providing ideal benchmarks for evaluating whether XAI methods can identify established drug scaffolds and understand chemical reasoning principles that have guided decades of medicinal chemistry optimization.

In AI drug discovery, molecular representation plays a crucial role in both the predictive accuracy and XAI ability, making careful selection critical [19–21]. Different representation strategies capture distinct aspects of molecular information and may influence how well AI models can predict and explain chemical relationships. We compared three distinct paradigms: molecular features and functional groups with classical Machine Learning, one-dimensional sequences of SMILES strings with Convolutional Neural Networks (CNN), and molecular graphs with Relational Graph Convolutional Networks (RGCN). While several other AI and XAI methods have been applied to small molecular drug discovery, they represent three fundamentally principles for representing molecular structures with one of the most common model architectures for each of the representations, providing a proof-of-concept study for comparing the explainability performance for fundamentally different molecular representations. Through systematic evaluation of these approaches against established structure–activity relationships in the three selected classes of antibiotics, we determine which provides the most reliable pathway for explainable AI in antimicrobial drug discovery.

## Methods

### Dataset preparation

For this study we used a dataset of molecular structures that have been evaluated for their growth inhibitory activity against the *Staphylococcus aureus*, a Gram-positive bacterial pathogen. The molecular structures were retrieved from ChEMBL (v34) [22, 23] together with their minimum inhibitory concentration (MIC) activity against *S. aureus*, with each compound categorized as either active (MIC ≤ 64 µg/mL) or inactive (MIC > 64 µg/mL). The dataset was further curated to ensure molecular validity and consistency by filtering compounds to exclude mixtures, inorganic compounds lacking carbon atoms, and molecules exceeding a molecular weight of 700 Da. The threshold of 700 Da is based on drug property guidelines for antibacterials, recommending more polar and larger molecules, compared to drug-likeness properties for other indications. This filtering process yielded a curated dataset of 43,777 unique chemical structures with 67% classed as actives and 33% as inactives. The distribution of chemical properties and chemical diversity is mostly uniform between actives and inactives, as shown in supplementary material Figures S1, S2 and S3, and Table S1.

The cleaned dataset was partitioned using stratified sampling into training (35,022 compounds, 80%), validation (4,378 compounds, 10%), and test sets (4,377 compounds, 10%) to preserve class proportions across all three modelling approaches.

### Training dataset and cross-validation

As outlined earlier, we implemented three distinct approaches in evaluating XAI capability: Classic Machine learning [24] methods using chemical feature representation, Convolutional Neural Networks using sequence-based SMILES representation, and Graph Convolutional Networks using molecular graph representation. Each model approach was trained using a 5 × 5 cross-validation strategy [25], of fivefold data cross-validation (5 variations of 4:1 split of data into training and validation set), each in 5 replicates using different seed numbers for weight initialisation, with all 25 trained model evaluated using the same 10% split test dataset. Model performance was evaluated using AUC_ROC_ (Area Under the Receiver Operating Characteristic curve), Precision, and Matthews Correlation Coefficient [26], using a prediction probability threshold of 0.5 across all replicates and models. While 5 × 5 cross-validation generated 25 models per approach, XAI analysis was done with only one of the replicates of the fivefold data cross-validation models.

All calculations were conducted using Python scripts together with RDKit [27] toolkit for any chemistry related calculations or manipulations, Scikit-Learn toolkit for machine learning and statistical calculations, and the PyTorch suite of PyTorch, PyTorch Lightning and PyTorch Geometry for GPU accelerated deep learning training and explainability applications.

### Molecular decomposition or fragmentation

To enable comparison of the explainability across different model architectures, different molecular representations and different explainability extraction methods, we used the same molecular fragments as common denominator to compare the different contribution scores. For this we applied BRICS (Breaking of Retro-synthetically Interesting Chemical Substructures) fragmentation [28] to generate a list of substructures for each molecule, and used model specific mapping methods to calculate for each substructure their contribution to the predictive activity scores. The BRICS fragmentation method identifies 16 retro-synthetically accessible bonds, that represent possible modification points which are amendable to chemical modification. Identifying bonds like alkyl-alkyl, alkyl-aromatic, aromatic-aromatic, alkyl-amine, aromatic-ether, or amide bonds, the resulting BRICS fragments also mimicking possible building blocks used in the synthesis of the compounds.

### Feature-based representation and model

For the first molecular representation we used chemical features and functional groups that are in line with the concept medicinal chemists use when describing structure–activity relationships, such as features like carbonyls, amines, imines, or large functional groups like imidazole. For this study we used the functional group definition in the RDKit toolkit, which defines 85 features and functional groups by the SMARTS substructure notation. As molecular descriptors we calculated a feature vector representing the counts of each of the 85 features present in a molecule. We selected this shorter denser feature vector over the sparser chemical fingerprint vector, with the intention to include a fragment-based descriptor with high, and most intrinsic interpretability.

These feature-based count vectors were further standardized, using StandardScaler (scikit-learn), to z-score normalize the counts across the whole dataset, before building a range of different machine learning classifier models to the predict antibiotic activity. Normalization is especially important to account for substantial variations in the occurrence of certain functional groups within a molecule (e.g., benzene rings typically appearing 0–3 times versus hydroxyl groups potentially occurring 0–15+ times).

For model selection we used LazyPredict [29] toolkit to automatically train and test 24 different classifier models. From these, Random Forest (RF) was selected as the classifier with the best performance across different key metrics (AUC_ROC_: 0.883, F1-Score: 0.828, MCC:0.585) (supplementary material Table S2). It is worth noting that no further finetuning was performed for the 24 different classifier models.

### Feature-based explainability

For explainability analysis, we employed TreeSHAP [8, 30] to evaluate feature contribution based on cooperative game theory. SHAP values quantify each feature's contribution to individual predictions by computing marginal contributions across all possible feature coalitions. For a decision-tree based prediction f(x), TreeSHAP decomposes the prediction into feature contributions:

$$f\left(x\right)={\upphi }_{0}+{\sum }_{i\in F}{\upphi }_{i}$$

where φ₀ is the baseline (expected prediction over the training data) and φ_i_ is the SHAP contribution value for feature *i*, representing its average marginal contribution across all possible feature combinations. We used a background dataset of 1,000 samples randomly selected from the training set to establish baseline feature distributions and calculated the SHAP values in probability space.

SHAP values are calculated for all 85 features, regardless of their presence in that given molecule. To map the feature contribution onto a molecule, we used the SMARTS pattern of each feature to map the corresponding SHAP values to all matching atoms, adding the values for multiple features matching the same atoms. These atom-based contribution scores are than used to calculate contribution scores for each BRICS fragment, by aggregation of the atom-based contribution scores of each atom in the fragment:

$${\mathrm{Contribution}}\left({\mathrm{fragment}}_{j}\right)={\sum }_{i\in {\mathrm{fragment}}_{j}}{\mathrm{Contribution}}\left({\mathrm{atom}}_{i}\right)$$

The resulting BRICS fragment contribution scores are nearly normal distributed, between −0.15 and +0.20 (see supplementary material Figure S4).

### Sequence-based representation and model

While feature-based approaches rely on predefined structural features, deep learning methods can learn relevant molecular patterns directly from chemical representations without any bias. Convolutional Neural Networks (CNN), originally developed for grid-topology processing in image recognition, were adapted to process molecular sequence data, by utilizing the SMILES notation of a molecular structure as one-dimensional sequences [31], allowing the model to discover complex molecular motifs and structural relationships that may not be captured by single functional group descriptors.

To enhance model robustness and prevent overfitting to specific SMILES representations, we employed SMILES augmentation by generating three different SMILES variants for each molecule using RDKit's randomized enumeration [32]. This augmentation provides different sequential representations of the same molecular structure, aimed to improve representation-invariant pattern recognition by any CNN. Each SMILES was then converted into a numerical vector using bag-of-word tokenizer, converting each character of a SMILES into one of the 76 separate tokens. The token vectors were further standardized to a maximum length of 181 by adding 0 as padding token and converted them into a binary input using one-hot encoding.

The CNN architecture implemented in PyTorch, contained as based model three sequential blocks, each consisting of Conv1D (with same padding to preserve sequence length), ReLU activation, and Dropout regularization, which progressively extract hierarchical chemical patterns while maintaining positional relationships within the molecular structure. Global average pooling then aggregates the spatial feature maps into a fixed-length molecular representation, creating a sequence-length-invariant descriptor. Finally, a linear layer maps this pooled representation to a binary classification output via Sigmoid activation.

**Fig. 1:**
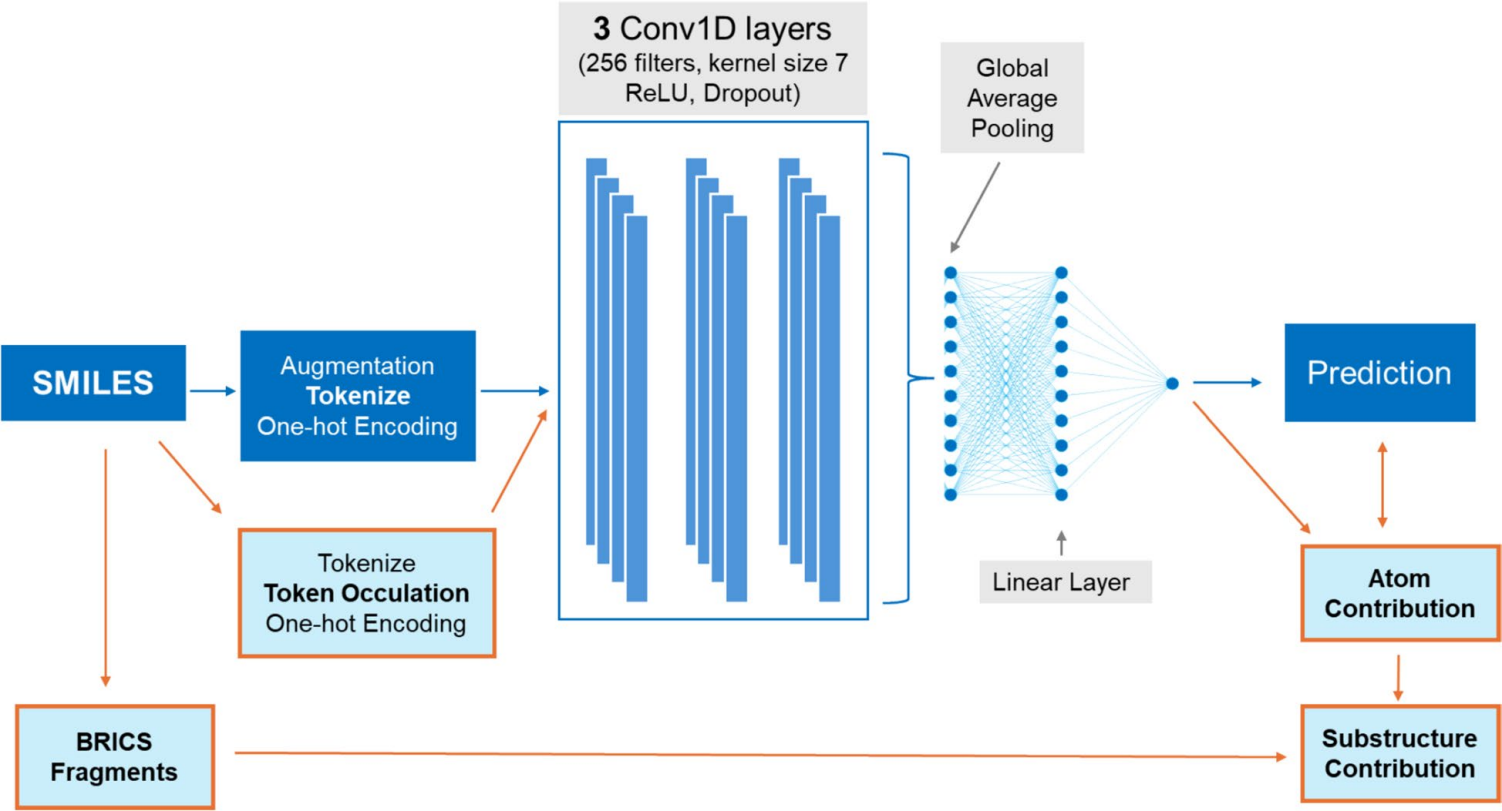
CNN architecture: Displaying in blue the prediction model, with tokenizing the SMILES sequence, conversion into one-hot-encoding, and input into the 3 layer convolutional layers, global average pooling into a final linear layer for prediction; In orange the explainability model, with sequential occlusion of the atom based tokens, calculating the difference between prediction and token-based prediction, and mapping of the atom-based contributions onto BRICS fragments for substructures-based contribution scores

Hyperparameter optimization employed Optuna's Tree-structured Parzen Estimator (TPE) across 50 trials [33], evaluating layer depth (1–3), filter counts (32–256), kernel sizes (3–7), dropout rates (0.1–0.5), and learning rates (1 × 10^–5^ to 5 × 10^–3^). TPE efficiently focuses on promising parameter combinations based on previous results rather than random sampling. Optimal performance (AUC_ROC_ = 0.828) was achieved with 3 layers, 256 filters with kernel size 7, and dropout rate of 0.199 with a total of 1.05M parameters (Fig. 1, supplementary material Table S4).

### Sequence-based explainability

For interpretability, we used a perturbation-based token masking (occlusion) method to obtain atom-level contribution scores [34, 35]. For this, each token representing an atom was systematically replaced with a padding token and prediction score calculated, and resulting change to the prediction score of the entire molecule recorded as token's attribution:

$${\mathrm{Attribution}}\left({\mathrm{token}}_{i}\right)=P\left({\mathrm{active}}|x\right)-P\left({\mathrm{active}}|{x}_{\setminus i}\right)$$

where x\i denotes the sequence with token *i* replaced by a padding token. Token attribution scores were mapped onto the corresponding atoms, aggregating the attribution scores for any elements (such as Cl and Br) with multiple using tokens. Each Similar to the previous method, these atom-based contribution scores were than used to calculate contribution scores for each BRICS fragment, by summation of the atom-based contribution scores of each atom in the fragment. The resulting BRICS fragment contribution scores are nearly normal distributed, between −0.97 and +0.96 (supplementary material Figure S4).

### Graph-based representation and model

While the SMILES notation represents a molecules’ graph structure with all its atomic connectivity’s and bond characteristics, the convolutional neural network treats the SMILES notation merely as a sequence of tokens, similar to transformer models in large language models (LLM), aiming to find patterns in those of sequences, A more ‘molecular’ pattern recognition approach is to use graph neural networks (GCN) on molecular graphs representations. Such approaches have been promising in predicting molecular properties, including antibacterial prediction [36].

For the GCN the molecular structure was represented as individual graphs, where atoms represent nodes with node features for element type, number of bonds, formal charge, hybridization, and aromaticity status. The node features were categorical encoded generating a 40 long node feature vector. Bonds were represented as edges with a single edge feature for bond type.

Relational Graph Convolutional Networks (RGCNs) are an extension to GCN able to handle multi-relational data through relation-specific transformations, explicitly modelling different edge types (or bond types) with separate weight matrices [37]. The "relational" component distinguishes RGCNs from other graph neural networks like direct message-passing neural networks (D-MPNNs) [38]. Recent studies have shown that graph-based models outperform other machine learning methods when working with sufficiently large datasets, making RGCNs a compelling choice for this comparative analysis [39, 40].

The RGCN architecture was based on the published RGCN implementation [37] which got adapted to PyTorch Geometric. It contained as based model two RGCNConv layers that aggregate neighbourhood information using bond-type-specific weight matrices, with each layer incorporating residual connections (via an additional linear transformation of the input), batch normalization, ReLU activation, and dropout regularization. The graph pooling stage employs weighted global sum aggregation, where learnable attention weights are computed per node via a sigmoid-activated linear transformation, and the final graph-level representation is obtained by summing the attention-weighted node features. The classification stage consists of three feed-forward blocks, each containing dropout, linear transformation, ReLU activation, and batch normalization, culminating in a final linear layer that maps the graph representation to a binary antibacterial activity prediction via sigmoid activation.

**Fig. 2:**
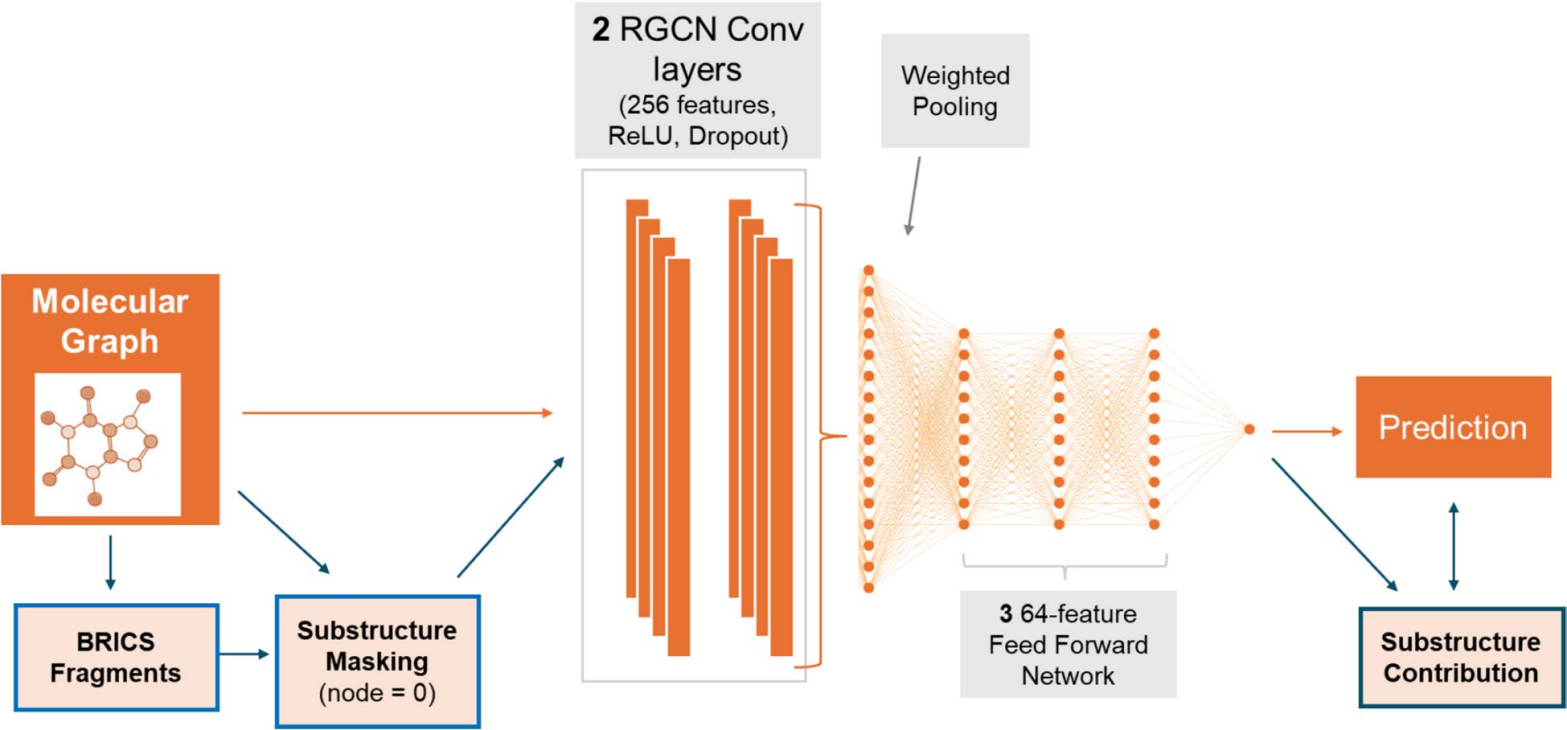
RGCN architecture: Displaying in orange the prediction model, with molecular graphs as input to the 2 RGCN convolutional layers, weighted pooling into the 3 feed forward layers for prediction; In blue the explainability model, with generation of BRICS fragments, sequential masking of the fragments in the molecular graph, calculating the difference between prediction and masked prediction for substructure-based contribution scores

Hyperparameter optimization was performed using Optuna's Tree-structured Parzen Estimator across 50 trials, exploring six different RGCN architectures (64–128, 128–256, 256–256, 64–64–128, 128–128–256, 64–128–256), two feed-forward network sizes (32, 64 features), five dropout levels (0.1–0.5) for both RGCN and feed-forward layers, and continuous ranges for learning rate (1 × 10^-4^ to 1 ×10^-2^) and weight decay (1 × 10^-6^ to 1 × 10^-3^). Optimal performance (AUC_ROC_ = 0.852) was achieved with 256–256 RGCN architecture, 64 feed-forward features, learning rate of 5.65 × 10^-4^, feed-forward dropout of 0.2, RGCN dropout of 0.1, and weight decay of 3.82 × 10^-4^ (Fig. 2, supplementary material Table S6).

### Graph-based explainability

For explainability, we employed a substructure-masking approach using the same BRICS decomposition [28, 41] method as before. Each substructure was systematically masked in the prediction of the activity probability score, by setting the node (or atom) feature vector to zero. The contribution score for each fragment was then calculated as difference in the predicted probability scores between the entire molecule and molecule with masked substructures:

$${\mathrm{Contribution}}\left({\mathrm{fragment}}_{j}\right)=P\left({\mathrm{active}}|x\right)-P\left({\mathrm{active}}|{x}_{{\mathrm{mask}}_{j}}\right)$$

where ${x}_{{mask}_{j}}$ denotes the molecular graph with all atoms in fragment_j_ having their atom features in the molecular graph set to a zero vector for masking. The resulting BRICS fragment contribution scores are nearly normal distributed, between -0.83 to + 0.93 (see supplementary material Figure S4). For visualisation of atom-based contribution all fragment-based contribution scores are mapped onto each of the atoms in the fragments using L1-normalisation across the atoms.

### XAI evaluation framework

#### Drug scaffold recognition

**Fig. 3:**
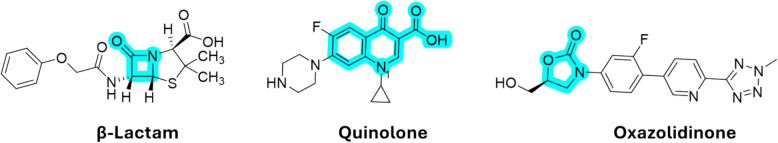
Reference molecules for antibiotic class evaluation with core scaffold highlighted in cyan. From left to right: Penicillin V (β-lactam class; β-lactam ring highlighted), Ciprofloxacin (fluoroquinolone class; bicyclic quinolone-carboxylic acid core highlighted), and Tedizolid (oxazolidinone class; oxazolidinone ring highlighted)

Scaffold recognition evaluates whether an explainability model can identify known drug scaffolds in active molecules as significant contributors to the prediction of its activity. For this we used the BRICS fragmentation method to identify all substructures in a molecule, determined their contribution score depending on the different model approaches (*i.e.* using the feature-based contribution score in RF, using the token-based attribution score in CNN or using the actual substructure-based contribution score in RGCN), and ranked them based on the contribution score. Next step was to identify if the drug core-scaffold for the different antibiotic classes (Fig. 3) was fully represented by the top 3 ranked BRICS substructures, using core-scaffold specific substructure searches (SMARTS):

**Table:** 

Class	Substructure	SMARTS	Structure
β-lactams [42, 43]	4-membered β-lactam ring	N1C(= O)CC1	
Quinolones [44]	bicyclic quinolone	c1ccc2ncccc2c1	
	free carboxylic acid	c[CX3](= O)[OX2H1]	
Oxazolidinone [45]	5-membered oxazolidinone ring	O1C(= O)NCC1	

For core-scaffolds which are split between multiple BRICS scaffolds, multiple SMARTS were defined with each SMARTS required to be represented in the top-ranked BRICS substructure list. For example, the quinolone core-scaffold had to be identified by both the bicyclic quinoline and a free carboxyl acid group.

#### Context sensitivity for activity cliff discrimination

The context sensitivity (CS) aims to evaluate if and how the explainability models can discriminate the active from the inactive molecule, within an activity cliff pair. For that we calculated two metrics: *Scaffold sensitivity* as percentage of activity cliffs in which the difference in contribution scores of the drug scaffolds is positive between active and inactive compounds; and *Substructure specificity* as the percentage of activity cliffs in which a substructure with negative contribution score is only present in the inactive molecule.

#### Model consistency between prediction and explanation

*Model consistency* between the prediction and the explainability models, was evaluated by the percentage of molecules where the mean of all fragment contribution scores was representative of the predicted activity, *i.e.* if the mean of contribution score was positive for predicted active molecules, or negative for predicted inactive molecules.

#### Model robustness

*Model robustness* evaluates the robustness of the explainability models across different instances of a model, *i.e.* different training dataset or different replicate. For this we calculated both the Pearson correlations [46] and Spearman correlations [47] of the fragment contribution scores for each molecule, between the 5 explainability model instances, one replicate model instances from each of the fivefold data cross-validation model instances. The final correlation values was computed as mean across all 10 pairwise model comparisons.

Pearson correlation [46] measures linear agreement on contribution score magnitudes across model instances:

$$r=\frac{\sum \left({x}_{i}-\overline{x }\right)\left({y}_{i}-\overline{y }\right)}{\sqrt{\sum {\left({x}_{i}-\overline{x }\right)}^{2}\sum {\left({y}_{i}-\overline{y }\right)}^{2}}}$$

where x_i_ and y_i_ represent contribution score for BRICS fragment i from two model instances, x̄ and ȳ are the mean contribution scores, and n is the number of matched fragments.

Spearman correlation [47] assesses consistency of fragment ranking across model instances:

$${\uprho }_{a,b}=1-\frac{6{\sum }_{i=1}^{n}{d}_{i}^{2}}{n\left({n}^{2}-1\right)}$$

where *d*_*i*_ represents the rank difference for BRICS fragment *i* between models *a* and *b*, *n* is the number of fragments being compared.

## Results and discussion

### Predictive models

We build predictive models using three different machine and deep learning model approaches, each with different molecular representation methods. We built each model using the same antibacterial activity dataset of molecules evaluated for their activity against *S. aureus*, a Gram-positive bacterial pathogen, and categorized as active by their minimum inhibitory concentration (MIC) in a dose–response growth inhibition assay against the bacteria. The dataset has been retrieved from ChEMBL [22, 23] a large online database for biological activity data. Actives were defined as compounds with an MIC ≤ 64 µg/mL, resulting in a dataset of 43,777 unique chemical structure with 67% classified as actives and 33% as inactives. This rather lenient threshold of 64 µg/mL for classifying actives represents the intended scope of the models, which is to identify any, even weakly active scaffolds and fragments, rather than identifying potential hits, for further drug development, where lower MIC thresholds of 4 or 2 µg/mL would be more appropriate. We also adopted a binary classification over regression, as regression models trained on -log(MIC) (pMIC) values tend to display weaker performance, due to the experimental variations in the MIC values, and the arbitrary definition of boundary values for inactives (i.e. MIC > 64 µg/mL) or highly actives (MIC ≤ 0.02 µg/mL).

The three different classification model included: *Random Forest* with feature-based representation (RF), Convolutional Neural Network models with sequence-based SMILES representation (CNN), and a Relational Graph Convolution Network with molecular graph-based representation (RGCN). For each approach the optimal model parameters were identified first: LazyPredict to identify Random Forest as the best performing model among 24 different machine learning methods (supplementary material Table S2), and hyperparameter optimisation to identify the best model parameter for the two deep learning methods CNN and RGCN (supplementary material Table S4 and S6). In the second step we used for each model type a 5 × 5 cross-validation [25] to evaluate model and data variations, using fivefold cross-validation of the training data (5 variations of 4:1 split of data into training and validation set), with 5 replicates using different seed numbers for initialisation (Table 1 and supplementary material Tables S3, S5, S7). The 25 predictive models for each approach were evaluated for their predictive performance using AUC_ROC_, Precision and Matthews Correlation Coefficient (MCC).

**Table 1:** Predictive performance metric for the three model approaches, as average and standard deviation, across the 25 training runs of the 5 × 5 cross-validation set, using the same test set

Method	AUC_ROC_	Precision	MCC
RF	0.866 ± 0.003	0.923 ± 0.002	0.545 ± 0.014
CNN	0.860 ± 0.015	0.918 ± 0.008	0.532 ± 0.035
RGCN	0.901 ± 0.068	0.947 ± 0.038	0.623 ± 0.150

All three approaches displayed similar acceptable performance for the prediction of the antibacterial activity against *S. aureus*, with RGCN displaying better performance across the performance metrics while also displaying variations in the cross-validations. It is worth noting, that the RF approach displayed a slightly better performance compared to the deep learning CNN model.

### Performance analysis of the Explainable AI models

We implemented a four-tier evaluation framework to assess the explainability performance of each approach. The four tiers represent increasing levels of interpretability performance: (1) *Scaffold Recognition* (SR), assessing the recognition of known drug scaffolds; (2) *Context Sensitivity* (CS), assessing explainability across activity cliffs; (3) *Model Consistency* (MC), assessing alignment between predictions and explanations; and (4) *Model Independence* (MI), assessing explainability consistency across different training conditions. These tiers form a structured basis for comparing model explainability performance together with its prediction performance.

### XAI Evaluation dataset

For the evaluation of the explainability performance we curated an evaluation dataset consisting of 300 activity cliffs (*i.e.* belonging to different experimental activity classification) calculated by matched molecular pairs (MMP) method in RDKit toolkit and filtered using a Tanimoto similarity threshold between the pairs of ≥ 0.5, (based on Morgan Fingerprints). In addition, the 300 activity cliffs were selected representing equally three of the most abundant antibiotic drug classes in the dataset: β-lactams, fluoroquinolones, and oxazolidinones (Fig. 3).

### Scaffold recognition (SR) for known drug scaffold

For the scaffold recognition we evaluated if the explainability method can identify the complete known drug scaffolds within active molecules, and ranked among the dominant, high-scoring substructures. For that we used the active molecules in the 300 activity cliff pairs of the XAI evaluation dataset and calculated the percentage of the unique compounds for which the drug scaffold was fully recognised (SR), together with the true positive rate (Recall) of the predictive model (Table 2).

**Table 2:** *Scaffold recognition* (SR) and *Recall* percentage among active molecules, by antibiotic classes and models

Antibiotic class	RF		CNN		RGCN	
	SR	Recall	SR	Recall	SR	Recall
β-lactam (n = 62)	98.4% (61)	100% (62)	98.4% (61)	91.9% (57)	100% (62)	100% (62)
Quinolones (n = 64)	98.4% (63)	100% (64)	70.3% (45)	59.4% (38)	85.9% (55)	98.4% (63)
Oxazolidinones (n = 11)	63.6% (7)	100% (11)	72.7% (8)	100% (11)	100% (11)	100% (11)
Total (n = 137)	95.6% (131)	100% (137)	80.3% (110)	80.3% (110)	93.4% (128)	99.3% (136)

The feature-based RF method achieved perfect prediction accuracy (100% recall) combined with excellent scaffold recognition (98.4%), demonstrating that simple chemical features descriptors can capture larger drug scaffold element. The RF approach only missed the scaffold recognition for 3 of the 64 quinolones, while still providing excellent prediction of those actives. The sequence-based CNN approach, on the hand, displayed quite some difficulty not only in recognising the drug scaffolds (80.3%) but also in the prediction accuracy of the 137 unique active molecules. Especially in the quinolone class, CNN was only able to recognize 70.3% of the complete drug scaffold, failing equally in scoring either the carboxylic group or the bicyclic quinoline within the top ranked substructures (Table 2). The CNN performed better for single fragment scaffolds, 98.4% and 100% for β-lactam and oxazolidinones, respectively, but still with low prediction recall rate of the actives. The graph-based RGCN method, on the hand, displayed good overall scaffold recognition (93.4%) and near perfect prediction accuracy, missing only one of the quinolone predictions. While displaying 100% rates for both β-lactam and oxazolidinones, it failed in the recognition of 10 of 64 complex quinolone drug scaffolds, even though it displayed near perfect activity prediction.

### Context sensitivity for activity cliff discrimination

Activity cliffs represent the most challenging scenarios for predictive models and XAI interpretation, as structurally similar molecules exhibit dramatically different biological activities, caused by only small chemical modifications. To evaluate the explainability behaviour of the different approaches, we used the set of 300 activity cliffs, 100 for each antibiotics class and calculated two explainability metrics, *Scaffold Sensitivity*: evaluating if activity cliffs can be explained by the different contribution scores of the two drug scaffolds itself, and *Substructure Specificity*: if a substructure other than the drug scaffold is able to explain the loss of activity, displaying a negative contribution score (Table 3).

**Table 3:** Scaffold Sensitivity and Substructure Specificity percentage among activity cliffs, by antibiotic classes and models

Antibiotic class	RF		CNN		RGCN	
	Scaffold sens	SubStr spec	Scaffold sens	SubStr spec	Scaffold sens	SubStr spec
β-lactam (n = 100)	94% (94)	59% (59)	71% (71)	50% (50)	72% (72)	57% (57)
Quinolones (n = 98*)	84.7% (83)	96.9% (95)	74.5% (73)	100% (98)	71.4% (70)	92.9% (91)
Oxazolidinones (n = 100)	96% (96)	87% (87)	80% (80)	82% (82)	63% (63)	88% (88)
Total (n = 298)	91.6% (273)	80.9% (241)	75.2% (224)	77.2% (230)	68.8% (205)	79.2% (236)

* 2 quinolones activity cliff excluded due to the lack of free carboxylate

The *Scaffold Sensitivity* rates were quite different between the feature-based RF approach and the two deep learning models, sequence-based CNN and graph-based RGCN. This might reflect the way the contribution scores are calculated by TreeSHAP method compared to both occlusion-based methods. TreeSHAP decomposes the prediction score into the different features, resulting in a stronger correlation of the individual feature contributions with the overall prediction score. In any case, contribution scores of the drug scaffold provide little value to a structure–activity relationship evaluation, since their structure does not change between the activity cliff pairs. They are also an indication on how much predictive ‘knowledge’ a model spends on an unmutable substructure, as a drug scaffold should have ideally similar contribution scores across both activity cliff pairs.

In contrast, the *Substructure Specificity* percentage is far more applicable to structure–activity relationship analysis, as it indicates how many activity cliffs can be explained by a single additional substructure. And surprising, all models display a similar behaviour, with similar behaviour within each antibiotic class. For β-lactams the models can identify in 50–57% of the activity cliffs a substructure responsible for the loss of activity, for oxazolidinones between 82–88%, and for quinolones between 93%-100%. The analysis also indicated that *Substructure Specificity* percentage is far more dependent on the antibiotic class, which might indicate bias from the nature of the drug scaffold definition, as the large quinolone drug scaffold allows more single substructure modifications, compared to the smaller drug scaffolds of β-lactams and oxazolidinones with more additional substructures having a more complex pattern of negative contributions.

### Model consistency between prediction and explanation

*Model consistency* is an important requirement for explainable model, with the explanations supporting their corresponding predictions [7, 48]. To evaluate the consistency between prediction and explainability, we used the 230 unique molecules from the 300 activity cliffs and evaluated if the mean of the contribution scores of all BRICS fragments in a molecule is positive for predicted actives or negative for predicted inactive (Table 4).

**Table 4:** Model consistency between prediction and explanation

Antibiotic class	RF	CNN	RGCN
β-lactam (n = 78)	94.9% (74)	91.0% (71)	94.9% (74)
Quinolones (n = 86)	93.0% (80)	89.5% (77)	96.5% (83)
Oxazolidinones (n = 66)	80.3% (53)	77.3% (51)	90.9% (83)
Total (n = 230)	90.0% (207)	86.5% (199)	94.3% (217)

All models demonstrated reasonably good consistency performance for most antibiotic classes, with graph-based RGCN at 94.3%, feature-based RF at 90%, and sequence-based CNN at 86.5% (Table 4). The graph-based RGCN approach was consistently outperforming the other approaches in all antibiotic classes, while the sequence-based CNN displayed the lowest consistencies in all antibiotic classes. While the consistency values do not reflect the quality of the prediction models, they do reflect the efficiency or coverage of the explainability methodology. RGCN displays the best consistency, by using the BRICS fragments to explain the contribution of those fragments directly while covering all atoms of the molecule. RF might lose some of the consistency due to a more general and overlapping mapping of the features contributions onto a molecule. And CNN might lose even more consistency by ignoring any special character tokens in the token occlusion method, relaying only on element tokens to calculate the contribution scores.

### Model robustness

The evaluate the internal robustness of the explainability models we evaluated the contribution scores between different model instances. For this we calculated the BRICS fragment contribution scores for molecules using the 5 different model instances from the cross-validation sets and analyse their pair-wise correlation, using a rank-based correlation (Spearman) and a value-based correlation (Pearson) coefficient (Table 5).

**Table 5:** Robustness of explainability model

Model	Pearson value-based correlation	Spearman rank-based correlation
RF	0.906	0.799
CNN	0.877	0.812
RGCN	0.839	0.696

All models display good value-based correlations (equal or above 0.8) between the contribution score from different instances of their models, with consistently better correlation in their values compared to their ranking (Table 5). Since all model displayed good consistency between expandability and prediction, these robustness metrics also reflect to general model uncertainties, which in this case might indicate some imperfect model training processes [49]. Graph Neural Networks are known for higher model complexity, requiring larger dataset compared to other models, to train models with similar generalisation performance [50]. The relational GCN adds even more model complexity by including node-edge relationships in the model. Indeed, RGCN is the model with the lowest XAI model robustness in this study, benefiting most to be training on a larger dataset.

### Activity cliff case studies

We performed a detailed analysis of some activity cliff cases, across three representative antibiotic classes, to investigate in more detail fragment-based contributions and their utility for medicinal chemists. The three pairs were selected based on structural similarity (Tanimoto ≥ 0.6) and significant differences in experimental activity, prioritizing true activity cliffs over prediction accuracy to test XAI capability.

Visualizations of the contribution scores employ a consistent colour scheme, with cyan indicating positive and orange negative contributions, und uncoloured regions indicating neutral contributions. Molecular structures were drawn in ChemDraw with manual colouring, with colour intensity reflecting the magnitude of the contribution score. The scale and distribution of contribution score differ slightly across the different approaches (supplementary material Figure S4) but are nearly normal distributed between -0.15 to +0.20, -0.97 to +0.96 and -0.83 to +0.93, for RF, CNN and RGCN, respectively.

### β-lactam activity cliff

**Fig. 4:**
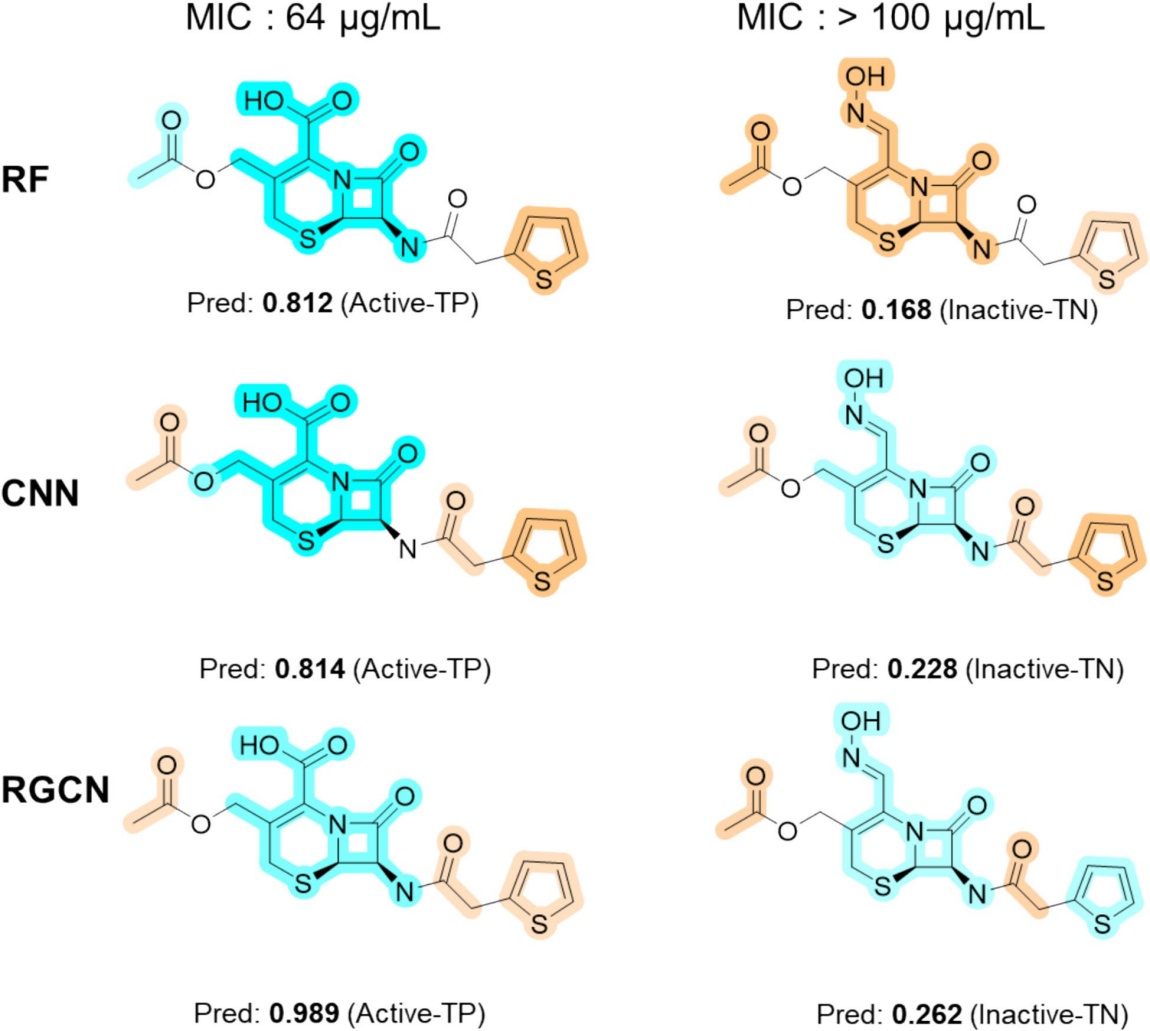
β-lactam activity cliff. Contribution patterns for active compound with carboxylic acid versus inactive with oxime. Cyan indicates positive contribution; orange indicates negative attribution. uncoloured regions reflect neutral contribution

The β-lactam activity cliff pair demonstrated a case when the structural modification or changes between the pairs is part of the core scaffold fragment (Fig. 4). The active compound (MIC: 64 µg/mL) contains a carboxylic acid, essential for activity, while the inactive compound (MIC: > 100 µg/mL) replaces this with an oxime, abolishing the negative charge required for target binding. All models achieved correct activity predictions for this pair.

RF shows the cephalosporin scaffold fragment with strong positive contribution for the active molecule (+0.182), correctly identifying the core drug scaffold. In the inactive molecule, the same scaffold, now containing the oxime, receives a negative contribution score (-0.098). On the other hand, in CNN and RGCN the same scaffolds receive positive contributions in both pairs, even though the magnitude is significant smaller in the inactive molecule, maintaining a correct discrimination between active and inactive, albeit shifted.

The other distinction between the models is the peripheral substructure contributions. CNN assigns strong negative contributions to the thiophene and acetyl substituent in both active and inactive, with very little discrimination, while RGCN shows negative contributions to the acetyl groups in both pairs and a wrong discrimination in the thiophene group.

This β-lactam activity cliff highlights one of the short comings of the applied decomposition method. The BRICS decomposition is unable to fragment bicyclic systems, leaving any cyclic substructure intact. In addition, it seems that most of the substituents of such cyclic systems are not cleaved either, resulting in this case, with one large fragment of the whole cephalosporin scaffold with either carboxylic acid or oxime for both active and inactive, respectively. With the current implementation of the fragment-based contribution this means that, any negative contribution of the oxime group will get averaged onto the positive cephalosporin scaffold. While the contribution score of the cephalosporin scaffold can discriminate between active and inactive, the contribution of the substituents of that cephalosporin scaffold are unfortunately obscured, independent of the quality of the explainability model.

### Quinolone activity cliff

**Fig. 5:**
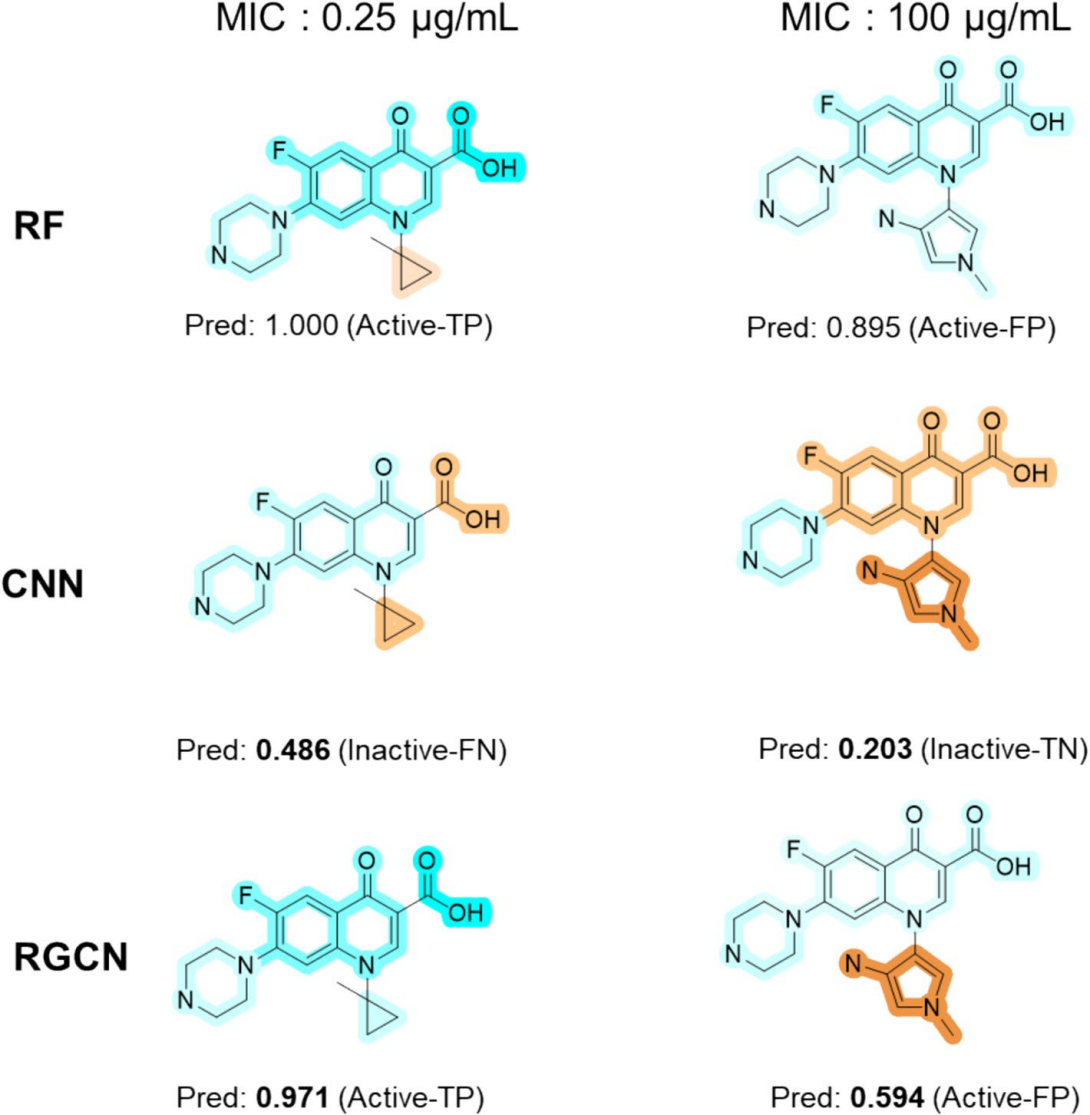
Quinolone activity cliff. Contribution patterns for active compound with cyclopropyl N1 substituent versus inactive with methylimidazol-2-amine N1 substituent. Prediction outcomes marked: FN (false negative), FP (false positive)

The quinolone activity cliff pair, on the other hand, has a different fragmentation pattern. While the bicyclic quinolone is one large fragment with fluorine, BRICS decomposes the carboxylic acid into a separate fragment (Fig. 5). This allows a better evaluation of the explainability between the different models. Indeed, the feature-based RF model struggles to characterize the inactive molecule correctly as inactive, even though the contribution score of the fluoro-quinolone scaffold is higher for the active molecule. One reason might be the lack of proper features to describe the methylimidazol-2-amine group, leaving that fragment with nearly neutral score. The sequence-based CNN has strong discrimination on the fluoro-quinolone scaffold, displaying negative contribution in the inactive molecule, while the carboxyl group which is essential for DNA gyrase inhibition through Mg^2^⁺ coordination, has a negative, contribution in both active and inactive molecule. Indeed, the CNN model struggled in correctly characterizing the active molecule as active. The graph-based RGCN model seemed to display the most rational contribution pattern with positive fluoro-quinolone scaffold and carboxylic acid in both active and inactive, with the only discriminatory contribution in the methylimidazol-2-amine, with strong negative contributions in the inactive molecule, even though RGCN struggles as well to characterize the inactive molecule as inactive. It seems that despite the strong negative imidazole group the overall prediction is carried by the large fluoro-quinolone scaffold and carboxyl group.

### Oxazolidinone activity cliff

**Fig. 6:**
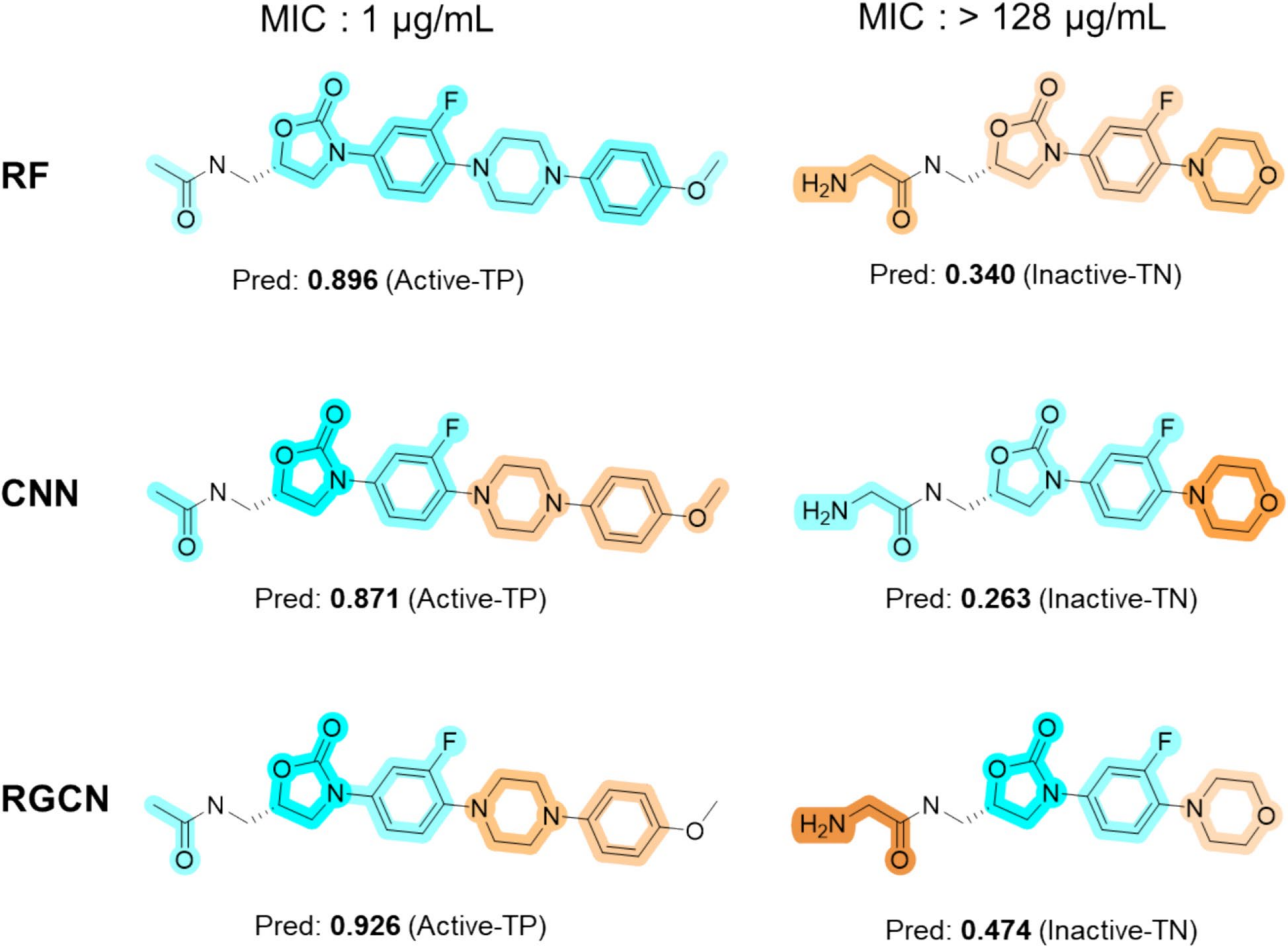
Oxazolidinone activity cliff: Contribution patterns for active compound with piperazine-acetamide versus inactive with morpholine-glycinamide

The oxazolidinone activity cliff pair is a case where there is no dominant large scaffold and the BRICS decomposition is able to provide an effective fragmentation into small cyclic substructures (Fig. 6). In addition, this activity cliff pair contains two structural changes, one on each side of the linear structure, providing a good test case of explainability.

All models are able to characterize the activity cliff pairs correctly, actives as actives, and inactives as inactives. For feature-based RF the fragment-based contributions scores are dominated by the overall prediction score, with all fragments positive in the active molecules, and all fragments negative in the inactive molecule, leaving little room for interpretation apart from a stronger negative contribution of the glycinamide group on the left-hand side. Both CNN and RGCN identify the oxazolidinone group as core positive contribution to the activity, together with the neighbouring 3-fluorophenyl group, both in active and inactive molecules. The 3-fluorophenyl group proved to be important for activity as well [51]. In addition, both identify the piperazine and phenolic group on the right-hand side as negative contributors in the active molecule, but not negative enough to shift the overall prediction. The right-hand side are regarded to not affect the activity and is sometimes used to attach groups for solubility or ADME properties. Similarly, the right-hand side of the molecule is scored negatively in the inactive molecule as well, with CNN putting the only and strong negative contribution on this morpholine group. On the other hand, the RGCN puts the discriminatory contribution mainly on the glycinamide group on the left-hand side, in better agreement with the current SAR studies.

## Limitations and future work

The scope of this study was to develop model independent performance metrics for XAI and evaluate their quality in a proof-of-concept study with three different approaches for predictive and explainable AI with three fundamentally different molecular representations. With the vast range of different AI technologies available to model chemical information, and with corresponding XAI technologies developing fast, it is worth mentioning some of the XAI approaches which have not been included in this study but could be applied to a fragment-based XAI evaluation. Such promising approaches include: SVM with SVERAD [52] or SVEKER [53], using a support vector machine on chemical fingerprints and Shapley values for explainability, or attention-based AI models, such as chemical language transformer models [54] or attention-based graph convolutional models [55].

A further property of the presented study is the relatively small size of the antibacterial dataset, limiting the application of larger deep learning models, such as attention-based transformer models. While there are some reservations on using attention-based approaches for explainability [56], larger transformer models are expected to capture long-distance relationships in SMILES better compared to CNN, but would also require larger, well balanced datasets for training. Such datasets are not always available for antibacterial activities, as high-throughput screenings have a very low hit-rate (1–2%) and are mostly not publicly available. Similarly, graph attention networks [57] would be worth to investigate, even though they have similar dataset requirements, with RGCN already showing some robustness limitations.

A final property highlighted in our activity cliff study is that the overall prediction of the activity is not always aligned with the overall fragment-based explainability, as illustrated in presented quinolone case for the RGCN approach, in which the overall prediction has a false positive while the explainability provides a better explanation for being inactive. While both are based on the same trained neural network and weights, their extraction of information (i.e. single probability score vs individual fragment contributions) allows for a slightly different interpretation of what the model learned. At this stage it is unclear if the model is just unable to assign to the negative affecting fragment a high enough importance (weight) to correctly predict it as inactive, and a larger dataset with more cases of the same fragment might help to learn it properly. What remains however, is the opportunity to investigate any false positive or negative for any fragment-based reasoning, even though such check would not be available when predicting the activity and fragment contribution for novel molecules.

## Conclusions

Using the data for antibiotic activity against *S. aureus*, a Gram-positive pathogen, we developed three different explainable AI models: Random Forest classifier using feature-based descriptors and SHAP values for explainability, Convolutional Neural Network using sequence-based SMILES descriptors with token occlusion for XAI, and Relational Graph Convolution Network using molecular graph descriptors and substructure masking.

To compare the explainability performance of the three XAI model, we established different evaluating matrices, utilizing fragment-based contribution scores as common denominator to compare XAI approaches with different molecular representations. The evaluation framework established 4 performance metrices: *Scaffold Recognition* (SR), assessing the recognition of known drug scaffolds; *Context Sensitivity* (CS), assessing explainability across activity cliffs; *Model Consistency* (MC), assessing alignment between predictions and explanations; and *Model Independence* (MI), assessing explainability consistency across different training conditions. In particular, the framework uses activity cliffs for performance evaluation, as active cliffs are particularly challenging for XAI to identify discriminatory features between very similar molecules.

All three antibacterial XAI models demonstrated strong predictive performance and good explainability property. Each XAI model was able to identify key drug scaffolds, displaying similar good model consistency and model independence. The main distinguishable performance indicators were in context sensitivity, in assessing positive and negative contributing fragments between activity cliffs pairs.

Random Forest with feature-based molecular representation was able to provide a good overall explainability performance with good scaffold recognition, but the contribution scores of the individual fragments were representing more the overall predictive score, rather than individual discrimination between fragments, providing the least utility for SAR interpretations. The CNN model with sequence-based SMILES also provided a good overall explainability performance with good scaffold recognition, while providing also a better discrimination between the different fragments, identifying fragment with more significant positive or negative contributions, and thereby providing a better utility for SAR interpretations. The token-occlusion method provided good explainability, but in some cases inconsistent with experiment SAR studies. The tokenizer methodologies is an important parameter for AI methods using sequence-based SMILES [58, 59], and in that sense different, more chemistry-aware tokenizer are might improve the accuracy of the explainability. The RGCN model with molecular graphs did have similar explainability performance as CNN, however identifying discriminatory fragments in activity cliffs in slightly better accordance with experimental SAR. The RGCN approach had however the lowest model robustness of the XAI models, which might improve by using a larger dataset for training.

The study also showed that while explainability model based on fragment-based contribution score are particularly suitable for SAR interpretation, they depend on the quality and behaviour of the molecular decomposition method. The BRICS decomposition provides chemically sensible fragmentation, but does not decompose multi-cyclic fragments, limiting the explainability outcome to larger fragments with averaged contribution score across the whole fragment. Different decomposition methods, like Murcko-derived leaf decomposition [33], or even multiple methods might provide a more comprehensive fragmentation pattern, including single cyclic system, substituents and linkers, with a more elaborate contribution scoring to account for overlapping fragments. The optimal selection of fragmentation patterns, which are able to provide an optimal SAR interpretation and utility to medicinal chemists, will mainly depend on the type of molecule, their chemical structure and their chemical synthesis, making it domain specific selection.

With the fast development of novel AI methods and availability of increasing larger datasets, chemoinformatic AI and XAI will become more and more a crucial corner stone into the drug development pipeline. And while no single AI architecture or single molecular representation or single XAI method will be optimal for the all the molecular and chemical diversity, it is essential to have a framework that can evaluate the quality, the limitation and the applicability of the different AI and XAI methods, allowing the medicinal chemistry to selected the most trustworthy and useful XAI system for their projects. The evaluation framework presented in this study is one of the such framework, applicable to a wide range of XAI technologies and to a wide range of property predictions.

## Supplementary Information

Additional file 1.

## Data Availability

The dataset, models and codes used for this work are available at [https://zenodo.org/records/18463329]. The repository contains notebooks to run molecules for each of the three models.
