# Supplementary material for: Framework for evaluating explainable AI in antimicrobial drug discovery
Source: J Cheminform. 2026 May 4;18:81. doi: 10.1186/s13321-026-01200-x (PMC13289538; doi:10.1186/s13321-026-01200-x)
Supplement: Supplementary file 1 — Additional file 1. [file 13321_2026_1200_MOESM1_ESM.docx]

**A Framework for Evaluating Explainable AI in Antimicrobial Drug Discovery**

Abdulmujeeb T. Onawole^1^, Mark A.T. Blaskovich^1,2^ and Johannes Zuegg^1^*

^1^Centre for Superbug Solutions, Institute for Molecular Bioscience, The University of Queensland, Brisbane, Queensland, Australia.

^2^ARC Centre for Agricultural and Environmental Solutions to Antimicrobial Resistance, Institute for Molecular Bioscience, The University of Queensland, Brisbane, Queensland, Australia.

*Correspondence to j.zuegg@uq.edu.au

**Supplementary Material**

[Datset preparation 2](#_Toc224549638)

[Training dataset and cross-validation 6](#_Toc224549639)

[Feature-based representation and model 6](#_Toc224549640)

[Sequence-based representation and model 8](#_Toc224549641)

[Graph-based representation and model 10](#_Toc224549642)

[Fragment-based explainability 12](#_Toc224549643)

# Datset preparation

**Table S1**: Molecular property statistics for active and inactive compounds in the *S. aureus* dataset (n=43,777). Six properties shown with numbers (N), Median, Min Max, and Mean: molecular weight (MW), partition coefficient (LogP), hydrogen bond donors (HBD), hydrogen bond acceptors (HBA), topological polar surface area (TPSA), and rotatable bond count.

| Property | Class | N | Median | Min | Max | Mean ± SD |
| --- | --- | --- | --- | --- | --- | --- |
| MW | Active | 29349 | 421.47 | 73.10 | 703.64 | 430.82 ± 106.26 |
|  | Inactive | 14428 | 385.42 | 58.08 | 703.27 | 394.48 ± 110.55 |
| LogP | Active | 29349 | 3.61 | -9.81 | 16.42 | 3.64 ± 2.22 |
|  | Inactive | 14428 | 3.57 | -10.19 | 14.79 | 3.55 ± 2.14 |
| HBD | Active | 29349 | 2 | 0 | 15 | 1.85 ± 1.59 |
|  | Inactive | 14428 | 1 | 0 | 15 | 1.63 ± 1.64 |
| HBA | Active | 29349 | 6 | 0 | 22 | 5.86 ± 2.64 |
|  | Inactive | 14428 | 5 | 0 | 19 | 5.39 ± 2.46 |
| TPSA | Active | 29349 | 86.99 | 0 | 389.61 | 90.95 ± 43.83 |
|  | Inactive | 14428 | 77.49 | 0 | 390.14 | 83.58 ± 42.31 |
| RotBonds | Active | 29349 | 5.00 | 0.00 | 41.00 | 5.93 ± 4.13 |
|  | Inactive | 14428 | 5.00 | 0.00 | 39.00 | 5.50 ± 3.97 |


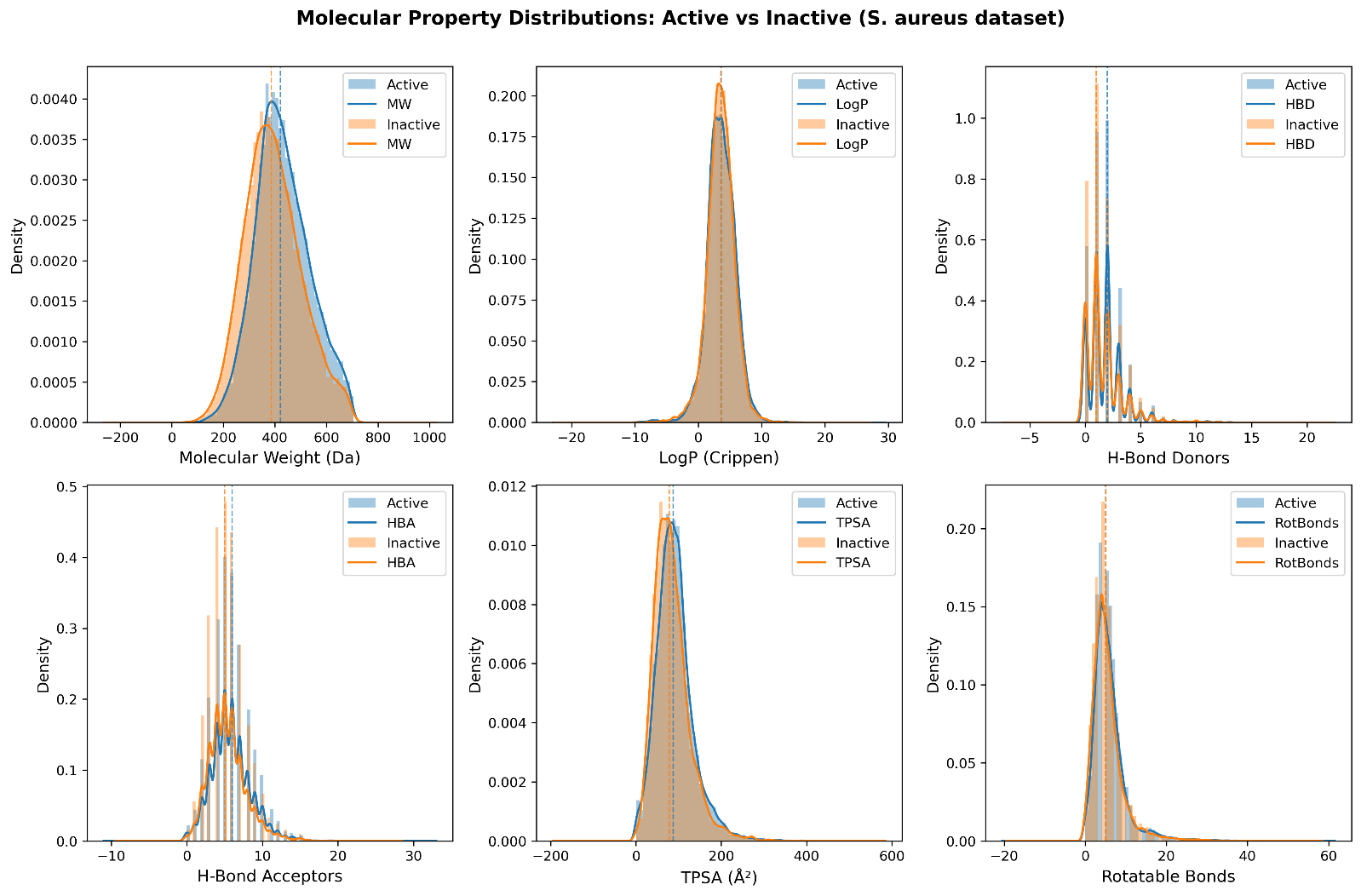


**Fig. S1**: Molecular property distributions for active (blue) and inactive (orange) compounds in the S. aureus dataset (n=43,777). Six properties shown: molecular weight (MW), partition coefficient (LogP), hydrogen bond donors (HBD), hydrogen bond acceptors (HBA), topological polar surface area (TPSA), and rotatable bond count. Distributions are shown as kernel density estimates.


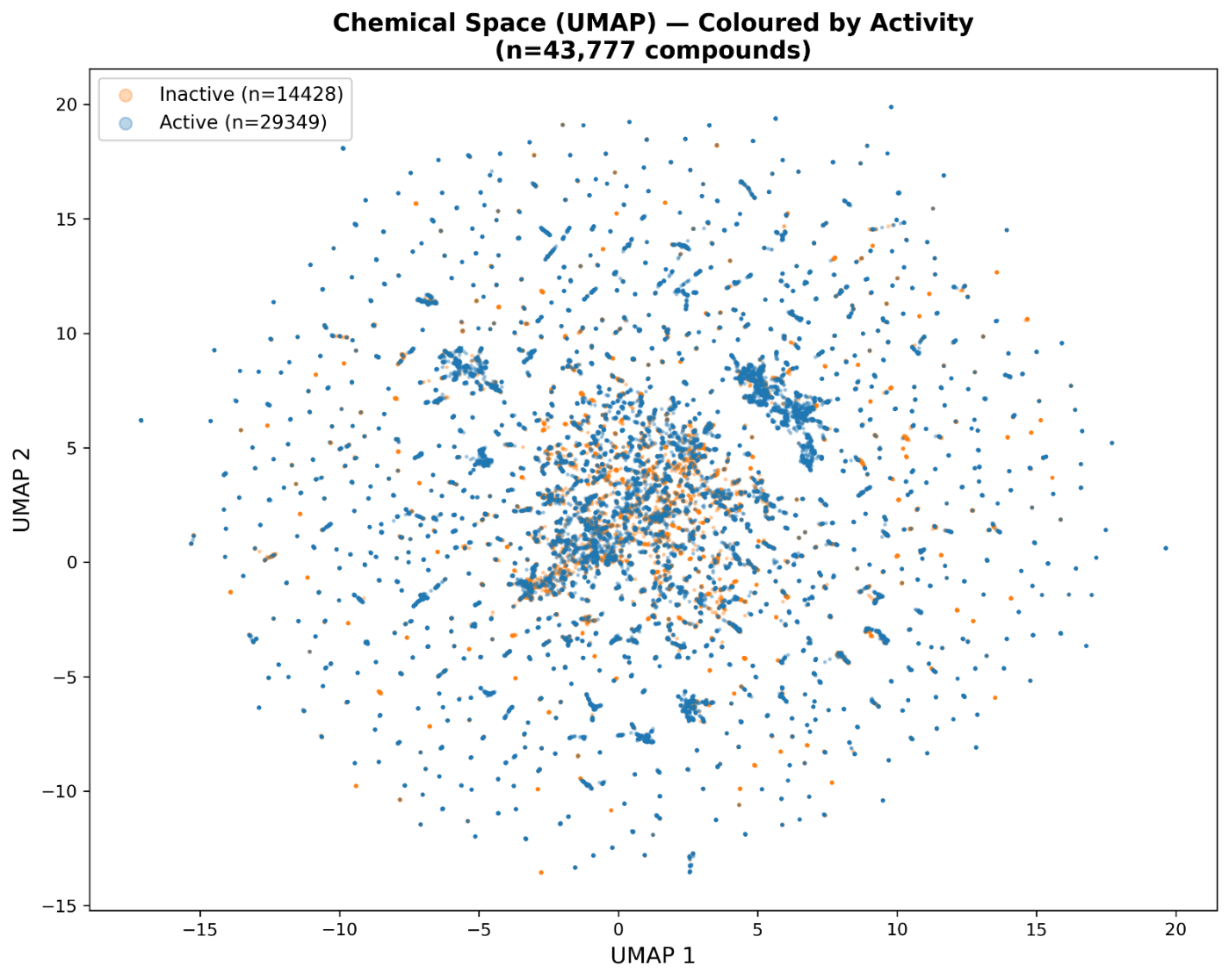


**Fig. S2:** UMAP projection of Morgan fingerprints (radius=2, 2048 bits, Jaccard metric) for all 43,777 compounds, coloured by activity label. Blue: active (MIC ≤ 64 µg/mL); orange: inactive (MIC > 64 µg/mL). Substantial intermixing throughout the chemical space is consistent with the prevalence of activity cliff pairs in this antibacterial dataset.


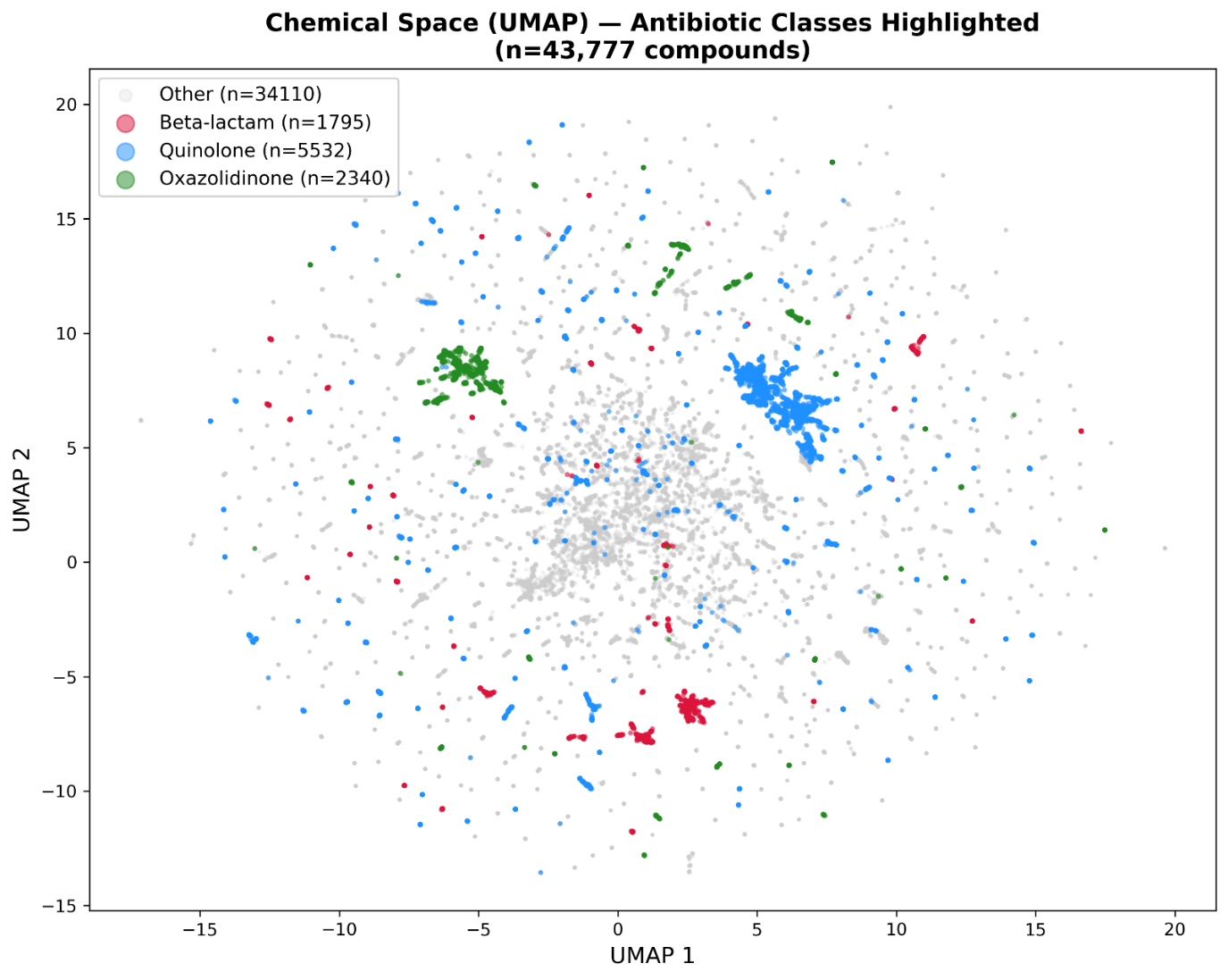


**Fig. S3:** UMAP projection (same embedding as Figure S2) with antibiotic class highlighted. Quinolones (n=5,532, red), oxazolidinones (n=2,340, green), and β-lactams (n=1,795, blue) form partially distinct structural clusters while retaining scattered members throughout broader chemical space**.**


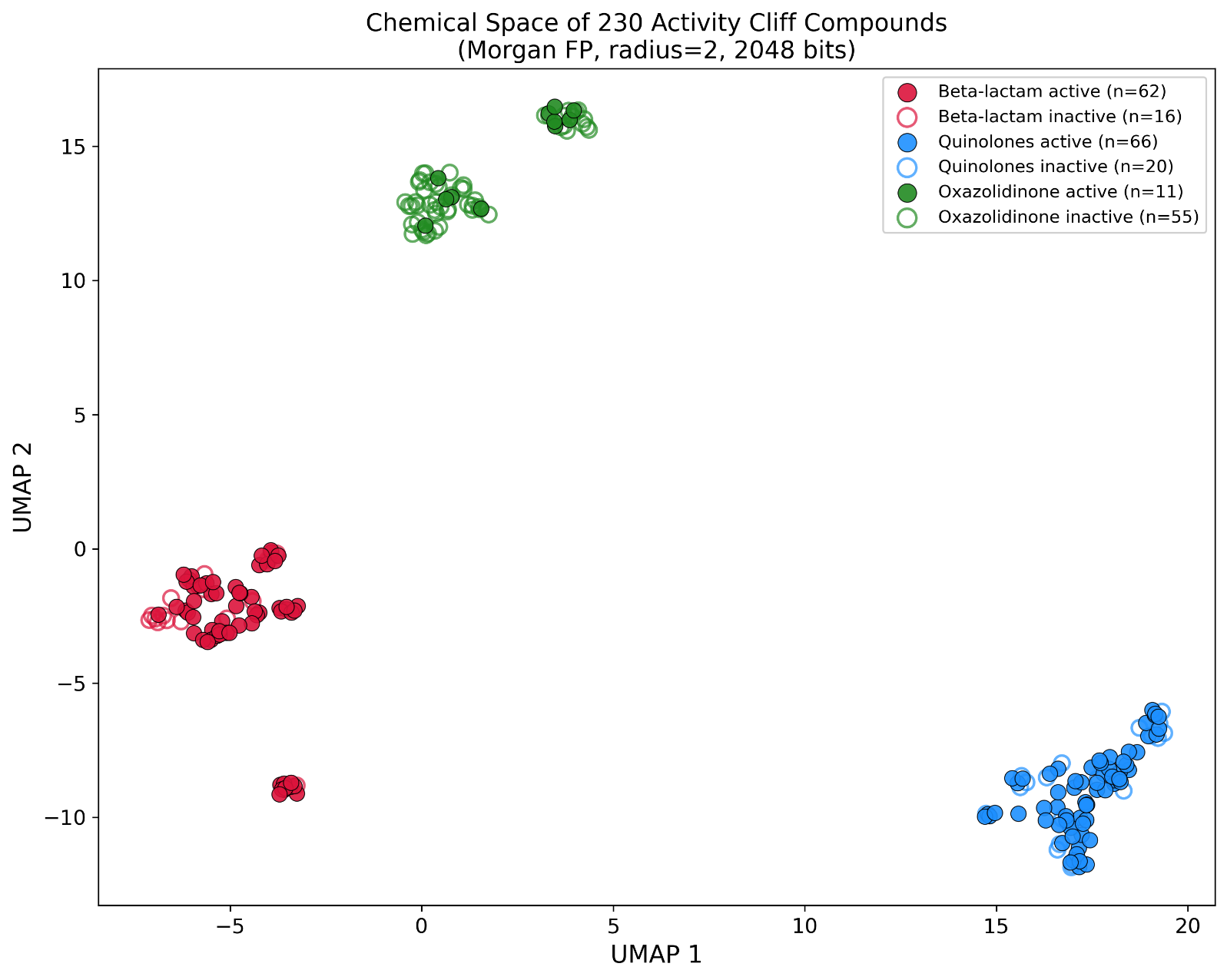


**Fig. S4:** UMAP projection of Morgan fingerprints (radius=2, 2048 bits, Jaccard metric) for the 230 unique compounds from 300 activity cliff pairs, coloured by antibiotic class (red: β-lactam; blue: quinolones; green: oxazolidinones) with filled markers for active compounds (MIC ≤ 64 µg/mL) and open markers for inactive analogues (MIC > 64 µg/mL). The three antibiotic classes form distinct clusters, confirming structural diversity across the evaluation dataset. Within each cluster, active and inactive compounds overlap extensively, consistent with the activity cliff design where paired compounds share the same scaffold but differ in activity due to peripheral substituent modifications.

# Training dataset and cross-validation

## Feature-based representation and model

**Table S2.** Performance comparison of 24 machine learning classifiers using LazyPredict for *S. aureus* antimicrobial activity classification.

| **Model** | **Accuracy** | **Balanced Accuracy** | **AUC_ROC_** | **F1 Score** | **MCC** |
| --- | --- | --- | --- | --- | --- |
| RandomForestClassifier | 0.820 | 0.783 | 0.883 | 0.869 | 0.585 |
| ExtraTreesClassifier | 0.817 | 0.788 | 0.865 | 0.865 | 0.583 |
| BaggingClassifier | 0.799 | 0.769 | 0.864 | 0.850 | 0.543 |
| KNeighborsClassifier | 0.786 | 0.743 | 0.833 | 0.845 | 0.504 |
| ExtraTreeClassifier | 0.777 | 0.752 | 0.762 | 0.832 | 0.501 |
| DecisionTreeClassifier | 0.776 | 0.753 | 0.762 | 0.830 | 0.500 |
| NuSVC | 0.782 | 0.721 | 0.834 | 0.847 | 0.484 |
| SVC | 0.748 | 0.660 | 0.804 | 0.830 | 0.390 |
| LGBMClassifier | 0.746 | 0.657 | 0.807 | 0.830 | 0.384 |
| GaussianNB | 0.480 | 0.585 | 0.637 | 0.410 | 0.196 |
| NearestCentroid | 0.564 | 0.601 | 0.601 | 0.600 | 0.193 |
| BernoulliNB | 0.629 | 0.597 | 0.640 | 0.714 | 0.188 |
| LogisticRegression | 0.679 | 0.561 | 0.689 | 0.792 | 0.174 |
| AdaBoostClassifier | 0.677 | 0.563 | 0.688 | 0.789 | 0.172 |
| CalibratedClassifierCV | 0.680 | 0.560 | 0.688 | 0.793 | 0.172 |
| LinearDiscriminantAnalysis | 0.679 | 0.558 | 0.688 | 0.793 | 0.169 |
| LinearSVC | 0.681 | 0.554 | 0.688 | 0.796 | 0.167 |
| RidgeClassifierCV | 0.682 | 0.552 | 0.688 | 0.798 | 0.167 |
| RidgeClassifier | 0.682 | 0.552 | 0.688 | 0.798 | 0.167 |
| SGDClassifier | 0.663 | 0.542 | 0.639 | 0.781 | 0.120 |
| Perceptron | 0.586 | 0.551 | 0.583 | 0.679 | 0.099 |
| QuadraticDiscriminantAnalysis | 0.368 | 0.521 | 0.641 | 0.120 | 0.089 |
| PassiveAggressiveClassifier | 0.586 | 0.517 | 0.530 | 0.700 | 0.036 |
| DummyClassifier | 0.668 | 0.500 | 0.500 | 0.801 | 0.000 |

**Table S3**. Test set evaluation of Random Forest models from 5×5 cross-validation (selected 5 models are in bold).

| **CV ID** | **AUC_ROC_** | **Precision** | **MCC** |
| --- | --- | --- | --- |
| CV1 R1 | 0.866 | 0.924 | 0.558 |
| CV1 R2 | 0.863 | 0.922 | 0.517 |
| CV1 R3 | 0.863 | 0.920 | 0.544 |
| **CV1 R4** | **0.868** | **0.924** | **0.553** |
| CV1 R5 | 0.867 | 0.921 | 0.559 |
| CV2 R1 | 0.868 | 0.922 | 0.553 |
| **CV2 R2** | **0.870** | **0.924** | **0.545** |
| CV2 R3 | 0.867 | 0.921 | 0.548 |
| CV2 R4 | 0.869 | 0.924 | 0.553 |
| CV2 R5 | 0.869 | 0.925 | 0.572 |
| CV3 R1 | 0.865 | 0.924 | 0.558 |
| **CV3 R2** | **0.869** | **0.924** | **0.549** |
| CV3 R3 | 0.862 | 0.918 | 0.559 |
| CV3 R4 | 0.865 | 0.923 | 0.536 |
| CV3 R5 | 0.865 | 0.920 | 0.553 |
| CV4 R1 | 0.865 | 0.920 | 0.529 |
| **CV4 R2** | **0.868** | **0.923** | **0.554** |
| CV4 R3 | 0.868 | 0.923 | 0.548 |
| CV4 R4 | 0.868 | 0.924 | 0.549 |
| CV4 R5 | 0.868 | 0.923 | 0.536 |
| CV5 R1 | 0.865 | 0.925 | 0.519 |
| CV5 R2 | 0.861 | 0.921 | 0.550 |
| CV5 R3 | 0.862 | 0.922 | 0.534 |
| CV5 R4 | 0.865 | 0.923 | 0.521 |
| **CV5 R5** | **0.866** | **0.924** | **0.534** |

## Sequence-based representation and model

**Table S4.** Hyperparameter optimization results for Convolutional Neural Network (CNN)

| **N** | **Layers** | **Filters** | **Kernel size** | **Dropout** | **Learning rate** | **Best epoch** | **Loss** | **AUC_ROC_** |
| --- | --- | --- | --- | --- | --- | --- | --- | --- |
| 0 | 2 | 32 | 3 | 0.1082 | 0.0041 | 5 | 0.412 | 0.748 |
| 1 | 1 | 128 | 5 | 0.2169 | 0.0001 | 100 | 0.419 | 0.690 |
| 2 | 2 | 256 | 7 | 0.4863 | 0.0015 | 89 | 0.359 | 0.807 |
| 3 | 3 | 128 | 3 | 0.2247 | 0.0003 | 100 | 0.373 | 0.779 |
| 4 | 3 | 64 | 3 | 0.1181 | 0.0001 | 100 | 0.402 | 0.726 |
| 5 | 3 | 128 | 7 | 0.4089 | 0.0000 | 100 | 0.380 | 0.771 |
| 6 | 2 | 64 | 5 | 0.2324 | 0.0000 | 100 | 0.418 | 0.691 |
| 7 | 3 | 64 | 5 | 0.4084 | 0.0002 | 100 | 0.376 | 0.774 |
| 8 | 1 | 128 | 5 | 0.2642 | 0.0011 | 100 | 0.399 | 0.734 |
| 9 | 3 | 64 | 3 | 0.4570 | 0.0003 | 100 | 0.397 | 0.736 |
| 10 | 2 | 256 | 7 | 0.3402 | 0.0045 | 95 | 0.360 | 0.805 |
| 11 | 2 | 256 | 7 | 0.3494 | 0.0049 | 100 | 0.359 | 0.800 |
| 12 | 2 | 256 | 7 | 0.4974 | 0.0015 | 95 | 0.356 | 0.811 |
| 13 | 2 | 256 | 7 | 0.4867 | 0.0011 | 81 | 0.358 | 0.811 |
| 14 | 2 | 256 | 7 | 0.4334 | 0.0010 | 55 | 0.358 | 0.809 |
| 15 | 2 | 256 | 7 | 0.4961 | 0.0006 | 100 | 0.358 | 0.808 |
| 16 | 1 | 32 | 7 | 0.3654 | 0.0019 | 100 | 0.395 | 0.740 |
| 17 | 2 | 256 | 7 | 0.4643 | 0.0005 | 100 | 0.359 | 0.807 |
| 18 | 2 | 256 | 7 | 0.3012 | 0.0023 | 74 | 0.363 | 0.812 |
| 19 | 2 | 256 | 7 | 0.1588 | 0.0026 | 74 | 0.381 | 0.807 |
| 20 | 1 | 32 | 7 | 0.2836 | 0.0006 | 100 | 0.408 | 0.717 |
| 21 | 2 | 256 | 7 | 0.3886 | 0.0025 | 83 | 0.365 | 0.806 |
| 22 | 2 | 256 | 7 | 0.3098 | 0.0012 | 92 | 0.375 | 0.809 |
| 23 | 2 | 256 | 7 | 0.4404 | 0.0008 | 100 | 0.360 | 0.812 |
| 24 | 2 | 256 | 7 | 0.4387 | 0.0007 | 100 | 0.361 | 0.806 |
| 25 | 2 | 256 | 7 | 0.1711 | 0.0004 | 85 | 0.373 | 0.808 |
| 26 | 2 | 256 | 7 | 0.3218 | 0.0001 | 100 | 0.365 | 0.794 |
| 27 | 2 | 256 | 7 | 0.4018 | 0.0024 | 89 | 0.359 | 0.810 |
| 28 | 1 | 32 | 5 | 0.4651 | 0.0004 | 100 | 0.416 | 0.698 |
| 29 | 2 | 32 | 3 | 0.3760 | 0.0031 | 74 | 0.402 | 0.728 |
| 30 | 2 | 256 | 3 | 0.4335 | 0.0009 | 87 | 0.388 | 0.751 |
| 31 | 2 | 256 | 7 | 0.4871 | 0.0017 | 100 | 0.359 | 0.810 |
| 32 | 2 | 256 | 7 | 0.4695 | 0.0015 | 100 | 0.359 | 0.810 |
| 33 | 2 | 256 | 7 | 0.4931 | 0.0033 | 100 | 0.355 | 0.807 |
| 34 | 2 | 128 | 7 | 0.4349 | 0.0009 | 100 | 0.360 | 0.801 |
| 35 | 2 | 256 | 7 | 0.4604 | 0.0019 | 100 | 0.361 | 0.810 |
| 36 | 3 | 256 | 7 | 0.2697 | 0.0014 | 100 | 0.384 | 0.820 |
| 37 | 3 | 128 | 5 | 0.2563 | 0.0001 | 100 | 0.377 | 0.774 |
| 38 | 3 | 64 | 3 | 0.2160 | 0.0008 | 100 | 0.381 | 0.771 |
| 39 | 3 | 256 | 7 | 0.1908 | 0.0035 | 87 | 0.392 | 0.812 |
| 40 | 3 | 64 | 5 | 0.1730 | 0.0033 | 100 | 0.360 | 0.802 |
| 41 | 3 | 256 | 7 | 0.2055 | 0.0012 | 100 | 0.409 | 0.820 |
| 42 | 3 | 256 | 7 | 0.2023 | 0.0013 | 63 | 0.415 | 0.815 |
| **43** | **3** | **256** | **7** | **0.1992** | **0.0002** | **72** | **0.402** | **0.828** |
| 44 | 3 | 256 | 7 | 0.1212 | 0.0002 | 70 | 0.434 | 0.826 |
| 45 | 3 | 128 | 7 | 0.1041 | 0.0002 | 89 | 0.393 | 0.823 |
| 46 | 3 | 128 | 3 | 0.1049 | 0.0002 | 100 | 0.377 | 0.780 |
| 47 | 3 | 128 | 7 | 0.1176 | 0.0001 | 76 | 0.362 | 0.785 |
| 48 | 3 | 128 | 7 | 0.1358 | 0.0001 | 100 | 0.386 | 0.815 |
| 49 | 3 | 128 | 7 | 0.1254 | 0.0000 | 100 | 0.368 | 0.788 |

**Table S5**. Test set evaluation of CNN models from 5×5 cross-validation (selected 5 models are in bold)

| **CV ID** | **AUC_ROC_** | **Precision** | **MCC** |
| --- | --- | --- | --- |
| CV1 R1 | 0.860 | 0.918 | 0.543 |
| CV1 R2 | 0.862 | 0.921 | 0.550 |
| CV1 R3 | 0.861 | 0.919 | 0.515 |
| CV1 R4 | 0.861 | 0.919 | 0.550 |
| **CV1 R5** | **0.869** | **0.924** | **0.552** |
| CV2 R1 | 0.864 | 0.920 | 0.526 |
| CV2 R2 | 0.862 | 0.919 | 0.547 |
| CV2 R3 | 0.864 | 0.922 | 0.540 |
| CV2 R4 | 0.863 | 0.920 | 0.533 |
| **CV2 R5** | **0.867** | **0.923** | **0.538** |
| **CV3 R1** | **0.867** | **0.923** | **0.547** |
| CV3 R2 | 0.864 | 0.920 | 0.543 |
| CV3 R3 | 0.854 | 0.916 | 0.505 |
| CV3 R4 | 0.790 | 0.880 | 0.376 |
| CV3 R5 | 0.864 | 0.919 | 0.539 |
| CV4 R1 | 0.860 | 0.918 | 0.540 |
| CV4 R2 | 0.862 | 0.920 | 0.537 |
| **CV4 R3** | **0.864** | **0.920** | **0.551** |
| CV4 R4 | 0.863 | 0.918 | 0.539 |
| CV4 R5 | 0.857 | 0.915 | 0.539 |
| CV5 R1 | 0.864 | 0.921 | 0.535 |
| CV5 R2 | 0.860 | 0.921 | 0.523 |
| CV5 R3 | 0.862 | 0.919 | 0.543 |
| **CV5 R4** | **0.865** | **0.923** | **0.530** |
| CV5 R5 | 0.862 | 0.918 | 0.563 |

## Graph-based representation and model

**Table S6**. Hyperparameter optimization for Relational Graph Convolutional Network (RGCN)

| **N** | **Learning rate** | **Weight decay** | **RGCN hidden  features** | **FFN hidden features** | **FFN dropout** | **RGCN dropout** | **Best epoch** | **Loss** | **AUC_ROC_** |
| --- | --- | --- | --- | --- | --- | --- | --- | --- | --- |
| 0 | 0.00073 | 0.00001 | 128-256 | 64 | 0.4 | 0.5 | 33 | 0.601 | 0.692 |
| 1 | 0.00234 | 0.00002 | 64-128-256 | 64 | 0.3 | 0.2 | 57 | 0.530 | 0.786 |
| 2 | 0.00012 | 0.00001 | 64-64-128 | 64 | 0.3 | 0.3 | 99 | 0.599 | 0.698 |
| 3 | 0.00052 | 0.00000 | 256-256 | 64 | 0.2 | 0.1 | 63 | 0.525 | 0.820 |
| 4 | 0.00011 | 0.00000 | 64-128 | 32 | 0.1 | 0.2 | 91 | 0.553 | 0.752 |
| 5 | 0.00610 | 0.00004 | 128-128-256 | 64 | 0.5 | 0.3 | 23 | 0.610 | 0.656 |
| 6 | 0.00053 | 0.00082 | 128-256 | 64 | 0.2 | 0.2 | 32 | 0.566 | 0.747 |
| 7 | 0.00030 | 0.00015 | 64-128 | 32 | 0.3 | 0.2 | 99 | 0.581 | 0.744 |
| 8 | 0.00747 | 0.00000 | 64-128-256 | 32 | 0.3 | 0.4 | 70 | 0.595 | 0.699 |
| 9 | 0.00595 | 0.00022 | 256-256 | 32 | 0.5 | 0.5 | 17 | 0.625 | 0.642 |
| 10 | 0.00191 | 0.00000 | 256-256 | 64 | 0.2 | 0.1 | 53 | 0.502 | 0.816 |
| 11 | 0.00159 | 0.00000 | 256-256 | 64 | 0.2 | 0.1 | 56 | 0.522 | 0.801 |
| 12 | 0.00164 | 0.00000 | 256-256 | 64 | 0.2 | 0.1 | 53 | 0.463 | 0.843 |
| 13 | 0.00035 | 0.00000 | 256-256 | 64 | 0.2 | 0.1 | 63 | 0.486 | 0.831 |
| 14 | 0.00030 | 0.00001 | 256-256 | 64 | 0.2 | 0.1 | 83 | 0.501 | 0.825 |
| 15 | 0.00026 | 0.00001 | 128-128-256 | 64 | 0.4 | 0.1 | 64 | 0.632 | 0.728 |
| 16 | 0.00024 | 0.00003 | 64-64-128 | 64 | 0.1 | 0.4 | 99 | 0.573 | 0.738 |
| 17 | 0.00041 | 0.00001 | 256-256 | 64 | 0.2 | 0.1 | 60 | 0.492 | 0.826 |
| 18 | 0.00019 | 0.00002 | 256-256 | 32 | 0.2 | 0.1 | 29 | 0.530 | 0.775 |
| 19 | 0.00092 | 0.00006 | 256-256 | 64 | 0.2 | 0.1 | 62 | 0.517 | 0.809 |
| 20 | 0.00016 | 0.00000 | 64-128 | 64 | 0.4 | 0.3 | 77 | 0.631 | 0.658 |
| 21 | 0.00039 | 0.00001 | 256-256 | 64 | 0.2 | 0.1 | 72 | 0.472 | 0.834 |
| 22 | 0.00041 | 0.00001 | 256-256 | 64 | 0.2 | 0.1 | 36 | 0.499 | 0.820 |
| 23 | 0.00072 | 0.00001 | 256-256 | 64 | 0.2 | 0.1 | 40 | 0.503 | 0.800 |
| 24 | 0.00035 | 0.00001 | 256-256 | 64 | 0.2 | 0.4 | 58 | 0.564 | 0.764 |
| 25 | 0.00020 | 0.00000 | 64-64-128 | 64 | 0.5 | 0.5 | 54 | 0.621 | 0.642 |
| 26 | 0.00054 | 0.00006 | 128-128-256 | 32 | 0.1 | 0.1 | 38 | 0.506 | 0.823 |
| 27 | 0.00112 | 0.00001 | 64-128-256 | 64 | 0.2 | 0.1 | 44 | 0.497 | 0.819 |
| 28 | 0.00023 | 0.00000 | 128-256 | 64 | 0.2 | 0.1 | 49 | 0.534 | 0.782 |
| 29 | 0.00312 | 0.00001 | 128-256 | 64 | 0.4 | 0.5 | 48 | 0.563 | 0.734 |
| 30 | 0.00084 | 0.00002 | 256-256 | 64 | 0.2 | 0.1 | 60 | 0.478 | 0.832 |
| 31 | 0.00041 | 0.00001 | 256-256 | 64 | 0.2 | 0.1 | 66 | 0.546 | 0.801 |
| 32 | 0.00062 | 0.00001 | 256-256 | 64 | 0.2 | 0.1 | 51 | 0.506 | 0.808 |
| 33 | 0.00015 | 0.00001 | 256-256 | 64 | 0.2 | 0.1 | 94 | 0.475 | 0.840 |
| 34 | 0.00036 | 0.00000 | 256-256 | 64 | 0.2 | 0.3 | 63 | 0.516 | 0.791 |
| 35 | 0.00030 | 0.00001 | 64-128-256 | 64 | 0.3 | 0.1 | 61 | 0.589 | 0.752 |
| 36 | 0.00118 | 0.00000 | 64-64-128 | 64 | 0.1 | 0.2 | 57 | 0.515 | 0.801 |
| 37 | 0.00044 | 0.00003 | 256-256 | 64 | 0.5 | 0.1 | 61 | 0.600 | 0.735 |
| 38 | 0.00011 | 0.00000 | 64-128 | 32 | 0.2 | 0.3 | 99 | 0.581 | 0.720 |
| 39 | 0.00065 | 0.00000 | 128-128-256 | 64 | 0.4 | 0.4 | 99 | 0.582 | 0.741 |
| 40 | 0.00015 | 0.00000 | 128-256 | 64 | 0.3 | 0.2 | 68 | 0.554 | 0.754 |
| 41 | 0.00048 | 0.00001 | 256-256 | 64 | 0.2 | 0.1 | 49 | 0.504 | 0.815 |
| 42 | 0.00033 | 0.00001 | 256-256 | 64 | 0.2 | 0.1 | 56 | 0.511 | 0.812 |
| 43 | 0.00041 | 0.00000 | 256-256 | 64 | 0.2 | 0.1 | 51 | 0.519 | 0.798 |
| 44 | 0.00026 | 0.00001 | 256-256 | 32 | 0.2 | 0.5 | 92 | 0.553 | 0.752 |
| **45** | **0.00057** | **0.00038** | **256-256** | **64** | **0.2** | **0.1** | **49** | **0.446** | **0.852** |
| 46 | 0.00021 | 0.00002 | 256-256 | 64 | 0.2 | 0.1 | 93 | 0.513 | 0.813 |
| 47 | 0.00020 | 0.00009 | 64-128-256 | 64 | 0.5 | 0.1 | 89 | 0.606 | 0.738 |
| 48 | 0.00013 | 0.00002 | 64-128 | 64 | 0.1 | 0.2 | 99 | 0.550 | 0.765 |
| 49 | 0.00029 | 0.00002 | 256-256 | 32 | 0.3 | 0.1 | 49 | 0.597 | 0.738 |

**Table S7**: Test set evaluation of RGCN models from 5×5 cross-validation (selected 5 models are in bold)

| **CV ID** | **AUC_ROC_** | **Precision** | **MCC** |
| --- | --- | --- | --- |
| **CV1 R1** | **0.966** | **0.983** | **0.766** |
| CV1 R2 | 0.908 | 0.953 | 0.607 |
| CV1 R3 | 0.876 | 0.934 | 0.554 |
| CV1 R4 | 0.829 | 0.908 | 0.474 |
| CV1 R5 | 0.939 | 0.969 | 0.690 |
| CV2 R1 | 0.831 | 0.909 | 0.464 |
| CV2 R2 | 0.768 | 0.870 | 0.371 |
| CV2 R3 | 0.839 | 0.916 | 0.480 |
| **CV2 R4** | **0.926** | **0.962** | **0.664** |
| CV2 R5 | 0.849 | 0.921 | 0.492 |
| CV3 R1 | 0.975 | 0.988 | 0.807 |
| **CV3 R2** | **0.982** | **0.991** | **0.831** |
| CV3 R3 | 0.792 | 0.886 | 0.409 |
| CV3 R4 | 0.899 | 0.947 | 0.607 |
| CV3 R5 | 0.974 | 0.987 | 0.794 |
| CV4 R1 | 0.931 | 0.964 | 0.674 |
| CV4 R2 | 0.947 | 0.973 | 0.711 |
| **CV4 R3** | **0.984** | **0.992** | **0.845** |
| CV4 R4 | 0.930 | 0.963 | 0.676 |
| CV4 R5 | 0.823 | 0.903 | 0.462 |
| CV5 R1 | 0.813 | 0.898 | 0.433 |
| **CV5 R2** | **0.980** | **0.990** | **0.819** |
| CV5 R3 | 0.847 | 0.918 | 0.485 |
| CV5 R4 | 0.964 | 0.982 | 0.762 |
| CV5 R5 | 0.946 | 0.973 | 0.705 |

# Fragment-based explainability


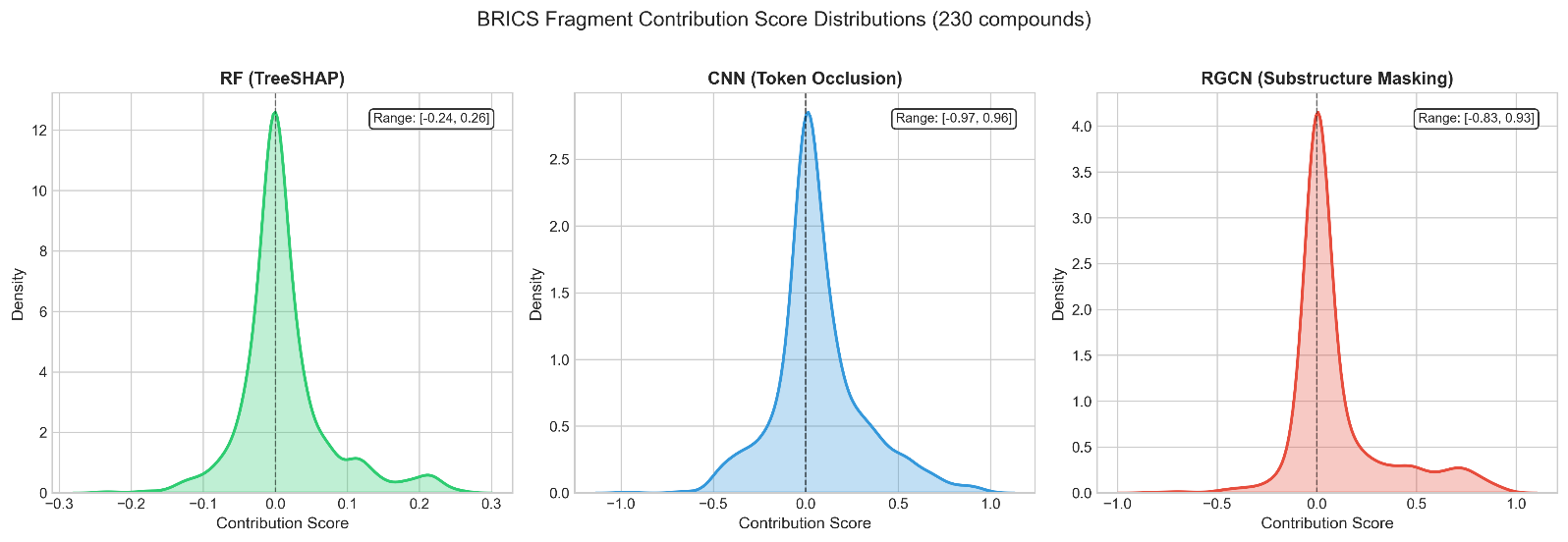


**Fig. S4**: Distrubtion of the BRICS fragment contribution scores for 230 unique compounds from the activity cliff, for the different models.

**Table S8**: Fragment number of compounds per antibtiotic class

| Antibiotic class | Fragment (n) | Actives | Inactives | Unique  Fragments | Common Fragments | Active only Fragments | Inactive only Fragments |
| --- | --- | --- | --- | --- | --- | --- | --- |
| Beta-lactam | 78 | 62 | 16 | 69 | 16 | 43 | 10 |
| Quinolones | 86 | 66 | 20 | 82 | 18 | 47 | 17 |
| Oxazolidinone | 66 | 11 | 55 | 69 | 14 | 8 | 47 |
| All | 230 | 139 | 91 | 186 | 40 | 83 | 63 |

Fragment (n): number of compounds per class. Unique Fragments: distinct BRICS fragments observed across all compounds in the class. Common Fragments: fragments present in at least one active and one inactive compound. Active-only / Inactive-only Fragments: fragments found exclusively in active (MIC ≤ 64 µg/mL) or inactive (MIC > 64 µg/mL) compounds respectively. BRICS fragmentation is deterministic and model-independent; identical fragments were confirmed across RF, CNN, and RGCN for all 230 compounds. The low proportion of common fragments (40/186, 21.5%) indicates that activity cliff pairs differ substantially in their peripheral substructures despite sharing the same core scaffold.
